# Reliability of Commercial Voice Assistants’ Responses to Health-Related Questions in Noncommunicable Disease Management: Factorial Experiment Assessing Response Rate and Source of Information

**DOI:** 10.2196/32161

**Published:** 2021-12-20

**Authors:** Caterina Bérubé, Zsolt Ferenc Kovacs, Elgar Fleisch, Tobias Kowatsch

**Affiliations:** 1 Centre for Digital Health Interventions Department of Management, Technology, and Economics ETH Zurich Zurich Switzerland; 2 Future Health Technologies Programme Campus for Research Excellence and Technological Enterprise (CREATE) Singapore-ETH Centre Singapore Singapore; 3 Centre for Digital Health Interventions Institute of Technology Management University of St. Gallen St. Gallen Switzerland

**Keywords:** voice assistants, conversational agents, health literacy, noncommunicable diseases, mobile phone, smart speaker, smart display, evaluation, protocol, assistant, agent, literacy, audio, health information, management, factorial, information source

## Abstract

**Background:**

Noncommunicable diseases (NCDs) constitute a burden on public health. These are best controlled through self-management practices, such as self-information. Fostering patients’ access to health-related information through efficient and accessible channels, such as commercial voice assistants (VAs), may support the patients’ ability to make health-related decisions and manage their chronic conditions.

**Objective:**

This study aims to evaluate the reliability of the most common VAs (ie, Amazon Alexa, Apple Siri, and Google Assistant) in responding to questions about management of the main NCD.

**Methods:**

We generated health-related questions based on frequently asked questions from health organization, government, medical nonprofit, and other recognized health-related websites about conditions associated with Alzheimer’s disease (AD), lung cancer (LCA), chronic obstructive pulmonary disease, diabetes mellitus (DM), cardiovascular disease, chronic kidney disease (CKD), and cerebrovascular accident (CVA). We then validated them with practicing medical specialists, selecting the 10 most frequent ones. Given the low average frequency of the AD-related questions, we excluded such questions. This resulted in a pool of 60 questions. We submitted the selected questions to VAs in a 3×3×6 fractional factorial design experiment with 3 developers (ie, Amazon, Apple, and Google), 3 modalities (ie, voice only, voice and display, display only), and 6 diseases. We assessed the rate of error-free voice responses and classified the web sources based on previous research (ie, expert, commercial, crowdsourced, or not stated).

**Results:**

Google showed the highest total response rate, followed by Amazon and Apple. Moreover, although Amazon and Apple showed a comparable response rate in both voice-and-display and voice-only modalities, Google showed a slightly higher response rate in voice only. The same pattern was observed for the rate of expert sources. When considering the response and expert source rate across diseases, we observed that although Google remained comparable, with a slight advantage for LCA and CKD, both Amazon and Apple showed the highest response rate for LCA. However, both Google and Apple showed most often expert sources for CVA, while Amazon did so for DM.

**Conclusions:**

Google showed the highest response rate and the highest rate of expert sources, leading to the conclusion that Google Assistant would be the most reliable tool in responding to questions about NCD management. However, the rate of expert sources differed across diseases. We urge health organizations to collaborate with Google, Amazon, and Apple to allow their VAs to consistently provide reliable answers to health-related questions on NCD management across the different diseases.

## Introduction

### Background

Noncommunicable diseases (NCDs) constitute a significant burden on public health [1,2] and are best controlled through self-management practice [3,4]. For this purpose, digital health technologies have been developed to assist individuals in managing disease in a scalable way and at a low cost [5]. Among other types of support [6], digital health tools can facilitate self-information on an on-demand basis (eg, decision support or information lookup devices).

### Voice Assistants for Information Lookup

Commercial voice assistants (VAs) can be employed on both smartphones and smart speakers, which are increasingly present in our daily lives. In fact, in 2018, 76% and 45% of individuals in advanced and emerging economies, respectively, owned a smartphone [7], and 66.4 million Americans owned a smart speaker [8]. Furthermore, although 148 million US adults used voice search for general purposes [9], 19.1 million used VAs for health-related purposes, such as gathering information about symptoms, medication, treatment options, or healthcare facilities [10]. Thus, VAs are not only scalable but also show considerable penetration.

In addition, VAs leverage speech-based interaction, which makes information lookup more efficient and accessible compared to classical methods (eg, desktop web search [11,12]). This is particularly the case in situations in which users have their hands occupied [13-17], lack reading and writing skills [18], or suffer from visual, motor, or cognitive inabilities [19-24] and cannot access information on display devices, such as smartphones, desktops, or tablets. Hence, VAs are a powerful alternative to provide chronic patients with easy access to information about NCD management. The question remains how well they can respond to health-related questions to facilitate NCD management.

### Related Work

Previous research investigated VAs’ reliability and assessed their ability to provide patients with reliable responses to health-related questions. These included mental and physical health, interpersonal violence [25], sexual health [26], smoking cessation [27], general health and lifestyle prompts [28], vaccines [29], addiction [30], postpartum depression [31], and COVID-19 [32]. The assessment methods varied across studies. Reliability was evaluated in terms of the ability to help with a safety-critical situation [25,28], the information correctness in comparison to official sources [31], the propensity to direct the user to available treatments or treatment referral services upon help seeking [30], or the reliability of the sources behind the responses [26,27,29,32]. All these studies have reported a rather poor performance, which is not surprising as this phenomenon has also been observed in other domains, such as shopping-related questions [9]. However, it is difficult to conclude which VA is the most reliable. For instance, although Google Assistant seems to be more reliable than Apple Siri for smoking cessation information delivery [27], the performance reverses for general lifestyle prompts [28]. Moreover, Google Assistant and Apple Siri are both more reliable than Amazon Alexa for vaccine-related questions [29]. Thus, it is unclear which VA best supports patients with chronic medical conditions in accessing information about NCD management.

### Objectives

This study evaluates the ability of common Vas, such as Amazon Alexa, Apple Siri, and Google Assistant, to vocally respond to questions related to NCD management, based on expert web sources. In particular, we measure reliability through *response rate* and *source type*. Based on previous research, we control for the effect of *developer* (ie, Amazon, Apple, and Google) and interaction *modality* (ie, voice-based interaction and multimodal interaction) [28] and compare the measures to web search results [27]. This comparison allows us to relativize the reliability of VAs to the standard consumer-accessible method of information retrieval. Conducting a web search may not always be the best solution, compared to consulting medical professionals [33], but patients are increasingly searching the internet for information [12]. Therefore, we consider web search as the method of reference for health information lookup.

Hence, we seek to answer the following research questions:

1. Is the *response rate* dependent on *developers* and *modality*?
2. Is the *source type* dependent on *developers* and *modality*?
3. Is the *response rate* dependent on *developers* and *disease*?
4. Is the *source type* dependent on *developers* and *disease*?

## Methods

Our experiment consisted of a quality assurance evaluation, where experimenters submitted validated questions to VAs and assessed (1) whether an error-free voice response was provided and (2) the category of the referenced web source.

### Research Design

We manipulated 3 independent variables: interaction *modality*, software *developer*, and *NCD*. We set a 3×3×6 fractional design with 3 *modalities* (ie, *voice only*, *voice and display*, *display only*), 3 *developers* (ie, *Amazon*, *Apple*, and *Google*), and 6 *disease*s (ie, *lung cancer [LCA], chronic obstructive pulmonary disease [COPD], diabetes mellitus [DM], cardiovascular disease [CVD], chronic kidney disease [CKD], and cerebrovascular accident [CVA]*). For the *display-only* modality, we solely included the developer *Google*. The dependent variables were the *response rate* (ie, percentage of provided voice responses) and the *source type* (ie, *expert*, *commercial*, *crowdsourced*, or *not stated*)*.*

### Measures

To measure the reliability of the different VAs, we assessed the *response rate* and *source type*. To assess the response rate, we computed the percentage of error-free speech-delivered responses. Specifically, a response was included if (1) the VA did not manifest an error, such as a system error (ie, a bug in the execution of a command), a natural language processing (NLP) error (ie, misunderstanding of the user’s utterance), or an intent error (ie, the user’s utterance is understood but leads to an unsupported command or an inappropriate execution), and (2) the response was voice-delivered without prompting the user to access information by interacting with a display device. For instance, if a VA was to reply *Here is what I found* and show results on the screen, we considered this response as not provided. Note that we leverage the use of VA for accessible, hands-free health-related information provision. Thus, we intentionally considered only those responses that could benefit individuals who cannot interact with a display.

In the *display-only* condition, we calculated the *response rate* by including all responses provided in the form of a web search featured snippet. A featured snippet is a unique box presented above the list of Google’s search results, containing a text answer to a question-like search query (what, how, when, etc). A snippet contains a title, a summary of the answer, and a link to the web source [34]. We chose this method to compare the ability of VAs to utter a selected response with that of the web search in pulling the best information source answering the question.

The source type was categorized based on the work of Alagha and Helbing [29] and Boyd and Wilson [27] into *expert*, *commercial*, *crowdsourced*, or *not stated*. Table 1 provides a description and examples of these categories. In particular, Alagha and Helbing [29] evaluated the VAs’ responses with points and categorized the web sources as *government*, *heath nonprofit*, *commercial*, *crowdsourced*, or *not stated*. If a web source was categorized as *government* and *heath nonprofit* (which they defined as *expert sources*), the response would gain a point and would otherwise get no points. Following this approach, we merged *government* and *heath nonprofit* categories into one that we called *expert* and consid*e*red such sources as *reliable* because they consistently assure good quality of information. Furthermore, we verified the reliability by exploring primary websites or by finding third-party sources of reliability evaluation (eg, Verywell Health is described as verified by doctors and collaborating with Cleveland Clinic, or Cancer.net presents patient information from the American Society of Clinical Oncology).

**Table 1:** Description of source types with examples.

Source type	Description	Examples
Expert	Site from a health organization representing a group of medical professionals, a governmental medical department or agency, a nonprofit medical organization, or a medical journal. These are considered reliable because they consistently provide verified and impartial information.	US Centers for Disease Control and Prevention (CDC), Mayo Clinic, *British Journal of Cancer*
Commercial	Any not nonprofit site that publishes medical information. These may or may not provide verified and impartial information.	WebMD, Medscape
Crowdsourced	Information from a site that is based on the collaboration of a large group of people. This information may or may not be verified and impartial.	Wikipedia, Blurtit
Not stated	The source type was not stated explicitly.	—^a^

^a^Not available.

### Selection of Health-Related Questions

Following Kvedar et al [5], we aimed to cover NCDs involving conditions considered the leading causes of mortality. Hence, we selected questions for Alzheimer’s disease (AD), LCA, COPD, DM (type 1 and type 2), CVD, CKD, and CVA. Regarding cancer, we decided on LCA as it is the most prevalent type of cancer affecting both sexes [35].

The questions were selected in 2 steps. First, we generated questions by collecting frequently asked questions (FAQs) from health organization, government, medical nonprofit, and other recognized health-related websites (eg, mayoclinic.org, nia.nih.gov, everydayhealth.com, webmd.com). When relevant, we looked for or replicated prevalent general questions across diseases. For instance, as we found the question *Is* *Alzheimer’s disease genetic?* on nia.nih.gov and *Is lung cancer genetic?* on foxchase.org, we searched for equivalents (eg, *Are strokes genetic?* on saebo.com) or arbitrarily created one for other diseases (eg, *Is chronic kidney disease genetic?*). This process allowed us to obtain a more comprehensive pool of questions.

Second, we asked practicing medical specialists to think about their medical consultations with patients suffering from the relative type of chronic disease and to rate the frequency of occurrence of the generated field-related question on a 5-point Likert scale (ie, 1=never, 2=rarely, 3=sometimes, 4=often, and 5=always). If they deemed it necessary, the specialists were also free to add manually missing frequent questions. For each disease, we recruited 2 specialists, resulting in a total set of 14 evaluations. We intentionally let the medical specialists rate questions in terms of absolute frequency and not ask, for instance, to indicate the *n most frequent questions*, as we wanted to avoid selection biases. The evaluations led us to an insufficient number of questions for AD. This can be explained by the fact that we asked the specialists to select questions that patients would ask their physician, and in the case of AD, information exchange rather happens between the physician and the caregiver, while the patient tends to be less involved [36]. Such tendency was confirmed by unsolicited comments from the specialists. Thus, following an intense discussion among the coauthors, we excluded the questions related to AD. Next, we included the 10 most frequent questions for all other diseases. If any resulted in a different formulation of the same question, only the question with the simplest formulation was included (eg, we favored *How long can I live with COPD?* over *What is the life expectancy for a COPD patient?*). If the ratings did not result in a clean cut of 10 questions, we defined further selection steps. If there were more than 10 questions, we favored wh-questions (ie, questions requiring an informative answer, rather than yes or no) and removed the most ambiguous questions (eg, we excluded *Why do I have difficulties breathing?* for COPD). If there were less than 10 questions, we included questions with lower ratings, always following the criteria mentioned above. Table 2 shows the number of questions included before and after the validation process (see the complete list of selected questions in Multimedia Appendix 1).

**Table 2:** Number of questions before validation from medical specialists.

NCD^a^	Questions generated (N=607), n (%)
AD^b^	48 (7.9)
LCA^c^	145 (23.9)
COPD^d^	60 (9.9)
DM^e^	135 (22.2)
CVD^f^	93 (15.3)
CKD^g^	69 (11.4)
CVA^h^	57 (9.4)

^a^NCD: noncommunicable disease.

^b^AD: Alzheimer’s disease.

^c^LCA: lung cancer.

^d^COPD: chronic obstructive pulmonary disease.

^e^DM: diabetes mellitus.

^f^CVD: cardiovascular disease.

^g^CKD: chronic kidney disease.

^h^CVA: cerebrovascular accident.

### Setting and Apparatus

The experiment took place in a meeting room at ETH Zurich, Switzerland, between February 18 and March 4, 2021. Each VA device was placed on a table and tested separately. Two experimenters sat at the two ends of the table. One experimenter submitted the questions to each device through a text-to-speech conversion program [37] on a laptop (Lenovo ThinkPad X1 Carbon Gen 8, Lenovo Group Limited). After pilot testing, we decided to use a female voice with a speech rate of 150 words per minute. For the questions to be adequately detected by the VAs, we played the questions through a set of 2 speakers (Sony Vaio VGP-SP1, VAIO Corporation) at a distance of ca. 10 cm from the VA device.

The other experimenter took note of the voice responses and the web source through either accessing them on the user accounts (smart speakers) or taking screenshots (display devices). To have a backup of the voice responses, we used an audio recorder (Philips DVT4010, Koninklijke Philips N.V.).

#### Tested VAs

Based on Kocaballi et al [28], we tested commonly used unimodal and multimodal VAs. To operationalize the variables *developer* and *modality*, we employed the 3 most common Vas (ie, Amazon Alexa, Apple Siri, and Google Assistant) and aimed for the 2 most frequently used devices (ie, smart speaker and smartphone) for each VA [10]. In particular, we used Amazon Echo Dot, Apple HomePod Mini, and Google Nest Mini for the *voice-only* modality and a mix of smart displays (Amazon Echo Show) and smartphones (Apple iPhone 8 with iOS 14.4, Nokia 6.1 with Android 9) for the *voice-and-display* modality. The latter heterogeneity in the devices was due to the unavailability of the Amazon Alexa smartphone application in the authors’ country of affiliation at the time of testing.

Moreover, to operationalize the *display-only* modality and our reference method of (health) information retrieval, we included a laptop with the Google Search engine (Lenovo ThinkPad X1 Carbon Gen 6 with Google Chrome). We referred to this condition as the *web search*.

All devices were set to factory settings, and we used new dedicated accounts, whose history was deleted before testing each device. Table 3 summarizes the implementation of the research design.

**Table 3:** Operationalization of the independent variables.

Modality	Amazon	Apple	Google
Voice only	Smart speaker (Eco Dot)	Smart speaker (HomePod Mini)	Smart speaker (Nest Mini)
Voice and display	Smart display (Eco Show)	Smartphone (iPhone 8, iOS 14.4)	Smartphone (Nokia 6.1, Android 9)
Display only	—^a^	—	Laptop (Lenovo ThinkPad X1 Carbon Gen 6, Google Chrome)

^a^Not available.

### Procedure

The selected questions were played in randomized order. In the *voice-only* and *voice-and-display* modalities, upon manual start, the text-to-speech program would play the appropriate wake-up keyword (ie, “Hey Alexa,” “Hey Siri,” or “Hey Google”), followed by a question. In the case of an error (see the Measures section), we first replayed the question and then, if necessary, played the question again using a male voice. In the case of a persistent error, 1 of the experimenters asked the question manually. This protocol allowed us to ensure the ability to respond to a question did not depend on the input quality. If none of those attempts produced a voice response, we considered the response as not provided. In the *display-only* modality, the question was directly entered in the text field of the web search engine.

### Ethics

Given the involvement of practicing medical specialists, we validated our research proposal (EK 2020-N-173) with the ETH Zurich Ethics Commission. The procedure was approved without reservation on December 21, 2020.

### Statistical Analysis

R Version 1.2 (RStudio, Inc.) was used to compute the frequency and descriptive statistics.

For the sake of comparison, all results (response rate and source type) are shown in percentages.

## Results

For each subsection, we first introduce the analysis and then present the results in the form of a table (see also Multimedia Appendix 2 for the complete list of voice responses, web sources, and categorization).

### Response Rate Across Developers and Modalities

To understand to what extent the examined VAs could provide an answer at all, we calculated the response rate across developers and modalities. The results are summarized in Table 4.

**Table 4:** Response rate across developers and modalities.

Response		Display only (N=60), n (%)	Voice and display (N=180), n (%)	Voice only (N=180), n (%)	Total (N=420), n (%)
**Amazon**					
	No	—^a^	14 (23.3)	15 (25)	29 (24.2)
	Yes	—	46 (76.7)	45 (75)	91 (75.8)
	Total	—	60 (100)	60 (100)	120 (100)
**Apple**					
	No	—	48 (80)	47 (78.3)	95 (79.2)
	Yes	—	12 (20)	13 (21.7)	25 (20.8)
	Total	—	60 (100)	60 (100)	120 (100)
**Google**					
	No	—	6 (10)	0	6 (5)
	Yes	—	54 (90)	60 (100)	114 (95)
	Total	—	60 (100)	60 (100)	120 (100)
**Web search**					
	No	12 (20)	—	—	12 (20)
	Yes	48 (80)	—	—	48 (80)
	Total	60 (100)	—	—	60 (100)

^a^Not available.

### Source Type Across Developers and Modalities

Classifying the web sources into *commercial*, *crowdsourced*, *expert*, and *not stated* allowed us to derive the level of reliability of the voice response across developers and modalities. The results are summarized in Table 5.

**Table 5:** Source type across developers and modalities.

Source type		Display only (N=48), n (%)		Voice and display (N=112), n (%)		Voice only (N=118), n (%)	Total (N=278), n (%)
**Amazon**							
	Commercial	—^a^	11 (23.9)		9 (20)		20 (22)
	Crowdsourced	—	4 (8.7)		4 (8.9)		8 (8.8)
	Expert	—	27 (58.7)		28 (62.2)		55 (60.4)
	Not stated	—	4 (8.7)		4 (8.9)		8 (8.8)
	Total	—	46 (100)		45 (100)		91 (100)
**Apple**							
	Commercial	—	2 (16.7)		2 (15.4)		4 (16)
	Crowdsourced	—	6 (50)		3 (23.1)		9 (36)
	Expert	—	1 (8.3)		1 (7.7)		2 (8)
	Not stated	—	3 (25)		7 (53.8)		10 (40)
	Total	—	12 (100)		13 (100)		25 (100)
**Google**							
	Commercial	—	12 (22.2)		12 (20)		24 (21.1)
	Crowdsourced	—	3 (5.6)		3 (5)		6 (5.3)
	Expert	—	39 (72.2)		45 (75)		84 (73.7)
	Total	—	54 (100)		60 (100)		114 (100)
**Web search**							
	Commercial	15 (31.3)	—		—		15 (31.3)
	Expert	33 (68.8)	—		—		33 (68.8)
	Total	48 (100)	—		—		48 (100)

^a^Not available.

Furthermore, we want to point out that all error-free responses involved the synthesis of a meaningful response and none resulted in the VA proposing to use a voice application to answer the question (eg, Alexa Skill or Google Action).

### Response Rate Across Developers and Diseases

We aimed to verify whether there was a pattern in the ability to provide an answer depending on the NCD in question. As the effect of modality was minimal, we present the results for the *voice-only* modality. Thus, we calculated the percentage of provided answers across *developer* and *disease*. Table 6 summarizes the results.

**Table 6:** Response rate by developer and disease.

Response		LCA^a^ (N=70), n (%)	COPD^b^ (N=70), n (%)	DM^c^ (N=70), n (%)	CVD^d^ (N=70), n (%)	CKD^e^ (N=70), n (%)	CVA^f^ (N=70), n (%)	Total (N=420), n (%)
**Amazon**								
	No	—^g^	8 (40)	2 (10)	6 (30)	7 (35)	6 (30)	29 (24.2)
	Yes	20 (100)	12 (60)	18 (90)	14 (70)	13 (65)	14 (70)	91 (75.8)
	Total	20 (100)	20 (100)	20 (100)	20 (100)	20 (100)	20 (100)	120 (100)
**Apple**								
	No	8 (40)	16 (80)	19 (95)	18 (90)	18 (90)	16 (80)	95 (79.2)
	Yes	12 (60)	4 (20)	1 (5)	2 (10)	2 (10)	4 (20)	25 (20.8)
	Total	20 (100)	20 (100)	20 (100)	20 (100)	20 (100)	20 (100)	120 (100)
**Google**								
	No	—	1 (5)	1 (5)	2 (10)	—	2 (10)	6 (5)
	Yes	20 (100)	19 (95)	19 (95)	18 (90)	20 (100)	18 (90)	114 (95)
	Total	20 (100)	20 (100)	20 (100)	20 (100)	20 (100)	20 (100)	120 (100)
**Web search**								
	No	1 (10)	1 (10)	1 (5)	3 (30)	5 (50)	1 (10)	12 (20)
	Yes	9 (90)	9 (90)	9 (45)	7 (70)	5 (50)	9 (90)	48 (80)
	Total	10 (100)	10 (100)	10 (100)	10 (100)	10 (100)	10 (100)	60 (100)

^a^LCA: lung cancer.

^b^COPD: chronic obstructive pulmonary disease.

^c^DM: diabetes mellitus.

^d^CVD: cardiovascular disease.

^e^CKD: chronic kidney disease.

^f^CVA: cerebrovascular accident.

^g^Not available.

### Source Type Across Developers and Diseases

Calculating the proportion of source types across developers allowed assessing the presence of information reliability patterns among the VAs. We present the results for the *voice-only* modality. Table 7 summarizes our results.

**Table 7:** Proportion of source type by developer and disease.

Source type			LCA^a^ (N=70), n (%)		COPD^b^ (N=70), n (%)		DM^c^ (N=70), n (%)		CVD^d^ (N=70), n (%)		CKD^e^ (N=70), n (%)		CVA^f^ (N=70), n (%)	Total (N=420), n (%)
**Amazon**														
	Commercial	2 (10)		4 (33.3)		6 (33.3)		3 (21.4)		3 (23.1)		2 (14.3)		20 (22)
	Crowdsourced	4 (20)		0		0		0		2 (15.4)		2 (14.3)		8 (8.8)
	Expert	10 (50)		6 (50)		12 (66.7)		9 (64.3)		8 (61.5)		10 (71.4)		55 (60.4)
	Not stated	4 (20)		2 (16.7)		0		2 (14.3)		0		0		8 (8.8)
	Total	20 (100)		12 (100)		18 (100)		14 (100)		13 (100)		14 (100)		91 (100)
**Apple**														
	Commercial	4 (33.3)		0		0		0		0		0		4 (16)
	Crowdsourced	5 (41.7)		0		0		1 (50)		1 (50)		2 (50)		9 (36)
	Expert	0		0		0		0		0		2 (50)		2 (8)
	Not stated	3 (25)		4 (100)		1 (100)		1 (50)		1 (50)		0		10 (40)
	Total	12 (100)		4 (100)		1 (100)		2 (100)		2 (100)		4 (100)		25 (100)
**Google**														
	Commercial	1 (5)		10 (52.6)		5 (26.3)		4 (22.2)		3 (15)		1 (5.6)		24 (21.1)
	Crowdsourced	4 (20)		0		0		0		2 (10)		0		6 (5.3)
	Expert	15 (75)		9 (47.4)		14 (73.7)		14 (77.8)		15 (75)		17 (94.4)		84 (73.7)
	Total	20 (100)		19 (100)		19 (100)		18 (100)		20 (100)		18 (100)		114 (100)
**Web search**														
	Commercial	2 (22.2)		5 (55.6)		4 (44.4)		1 (14.3)		2 (40)		1 (11.1)		15 (31.3)
	Expert	7 (77.8)		4 (44.4)		5 (55.6)		6 (85.7)		3 (60)		8 (88.9)		33 (68.8)
	Total	9 (100)		9 (100)		9 (100)		7 (100)		5 (100)		9 (100)		48 (100)

^a^LCA: lung cancer.

^b^COPD: chronic obstructive pulmonary disease.

^c^DM: diabetes mellitus.

^d^CVD: cardiovascular disease.

^e^CKD: chronic kidney disease.

^f^CVA: cerebrovascular accident.

## Discussion

### Principal Results

Google showed the highest response rate and rate of expert sources, leading to the conclusion that Google Assistant would be the most reliable tool in responding to questions about NCD management. However, the rate of expert sources differed across diseases.

#### Response Rate Across Developers and Modalities

We observed Google Assistant provided the highest response rate, even outperforming the web search results. Apple Siri showed the lowest response rate. This specific advantage of Google Assistant is consistent with previous studies [27,29,32,38,39].

Moreover, Apple Siri often replied *I found this on the web* and presented visually a list of results instead of *voicing* a unique response. This may reflect a tendency to transfer the responsibility of information retrieval to the patients, whereas they are in charge of choosing (the most) reliable information source. As we believe in the potential of VAs in increasing the accessibility of health information by *vocally* interacting with patients having their hands occupied or with disabilities [13-17,19-24], we urge Apple to consider favoring voice responses over lists of results to make the information search more suitable for hands-free interaction.

#### Source Type Across Developers and Modalities

Similar to the results for the response rate, Google Assistant also answered the most frequently with *expert* sources, without outperforming the web search results. Amazon Alexa showed 13.3% fewer *expert* sources. These results are surprising, given the partnership that Amazon started with the United Kingdom National Health Service (NHS) in 2019, allowing the former to freely access health-related information provided by the latter [40]. Finally, Apple Siri showed the least *expert* sources, favoring *crowdsourced* sources. Importantly, Apple Siri also showed the highest proportion of missing sources (ie, *not stated*). Alagha and Helbing [29] classified *commercial*, *crowdsourced*, and *not stated* sources as less reliable than *expert* sources. As *commercial* and *crowdsourced* sources may, in some cases, still provide reliable information [41,42], Apple Siri may tend to transfer the responsibility of judging the reliability of the information to the patient. This assumption is also supported by the low rate of voice responses from Apple Siri discussed above.

#### Response Rate Across Developers and Diseases

When comparing the response rate across diseases, we observed Google Assistant to respond rather similarly across diseases, with a slight advantage for LCA and CKD. Amazon Alexa and Apple Siri showed the highest response rate for LCA. Thus, questions related to LCA seemed to have a general advantage over the other diseases. Both Amazon Alexa and Google Assistant outperformed web search results on both LCA and CKD.

#### Source Type Across Developers and Diseases

Our results showed Google Assistant to most often use *expert* sources for CVA, while Amazon Alexa did so for DM. Apple Siri showed *expert* sources only for questions about CVA. Thus, there seems to be an advantage of CVA questions in being reliable the most often, except when those questions are asked to Amazon Alexa.

Finally, this slight heterogeneity in results is in line with the diversity in previous research, whereas depending on the condition or disease of interest, reliability varies across VAs [27-29]. Hence, there seems to be a need for systematization of health information search algorithms across the different medical domains.

### Limitations

Despite our best efforts, our study presents some limitations.

First, for technical reasons, we employed a smart display instead of a smartphone for Amazon Alexa. The observed similarity in source type proportion between modalities may be explained by the use of 2 types of home devices. This similarity may reflect a consistency in information provision across Amazon Alexa modalities, which is desirable. However, future research should aim to replicate the results by comparing the reliability between the Amazon Alexa app and an Amazon smart speaker. This will support an absence of the effect of interaction modality in Amazon Alexa.

Second, to control for the effect of time on the responses, we aimed to restrict the time window inside which to test the VAs as much as possible. Thus, given the high number of questions submitted (ie, 60 questions per device, resulting in a total of 420 submissions), we did not submit the same questions multiple times. However, we conducted a post hoc test-retest reliability assessment by randomly selecting 1 question per NCD and submitting each one 10 times to all VAs. Our results showed no variation in the voice responses or the source type. Nevertheless, as observed in previous research that VAs do not always provide the same response [29], future research should consider the test-retest reliability of VAs in responding to a more extensive set of questions about the included NCDs.

Third, questions were selected by looking for FAQ pages on health-related websites, but it is difficult to conclude whether all relevant questions were included. As we shared the list of questions (see Multimedia Appendix 1), we hope future research will be able to establish whether additional relevant questions need to be tested.

Fourth, the source type was categorized based on the work of Alagha and Helbing [29] and Boyd and Wilson [27] into *expert*, *commercial*, *crowdsourced*, or *not stated*, whereas *expert* sources represented *government* and *heath nonprofit* sources [29]. We consid*e*red such sources as the *most* *reliable* because they consistently assured good quality of information. However, although not stating a source of information makes it difficult for a patient to judge the response’s trustworthiness, *commercial* and *crowdsourced* sources may, in some cases, still provide correct information. Health-related information coming, for instance, from wikipedia.com (*crowdsourced*) varies importantly in terms of quality [41] and could, in some cases, still provide reliable information. *Commercial* sources may also contain partly reliable information [42], despite presenting a higher risk of bias toward marketing purposes [43]. Future research should investigate directly the reliability of the provided information rather than the mere source type in order to have a more fine-grained landscape of its reliability.

Fifth, the FAQs about AD were evaluated as rather rarely asked by the patients. As information exchange about AD rather happens between the physician and the caregiver, while the patient tends to be less involved [36], future research should include questions from caregivers as well in order to assess the response and *expert* source rates for FAQs related to this disease.

Finally, questions were validated by Swiss doctors and thus may not be representative of the patient’s most frequent concerns in other realities. Future research should validate the tested questions with professionals of other countries to ensure their relevance across nations.

### Comparison With Related Work

#### Comparing VAs to Web Search

Our results are partly in line with the work of Boyd and Wilson [27], comparing the ability of Apple Siri and Google Assistant (*voice-and-display* modality only) to provide expert information for smoking-cessation-related questions compared to the web search. Similar to Boyd and Wilson [27], we observed Google Assistant to be more reliable than Apple Siri at providing information from reliable sources (which in their study was defined as web pages of *health agencies with medical expertise*). However, although Boyd and Wilson [27] found the web search to be more reliable better than Google Assistant, our evaluation showed Google Assistant to be slightly more reliable than the web search (in both *voice-and-display* and *voice-only* modalities). The difference may lie in the evaluation of the web search’s responses: although we considered featured snippets as the selected response to judge the search engine on, Boyd and Wilson [27] considered the *first non-advertisement link or information* of the list of results. This criterion may have led the authors to collect more responses from the web search and thus a different distribution of reliable sources. Moreover, Boyd and Wilson [27] were the only ones comparing the reliability of the VAs to a traditional method of health information search, such as browser-based web search. Not comparing the VAs' response rate and information reliability to web search makes it difficult to conclude on their absolute ability to respond to health-related questions. Our results not only show Google Assistant leading in NCD-related information provision, but also that it can even outperform the web search results and quite successfully inform patients about NCD management.

#### Evaluating Response Source Type

Based on Alagha and Helbing [29], who based their evaluation system on Boyd and Wilson [27], we evaluated the source type and classified the sources as *not stated*, *crowdsourced*, *commercial*, or *expert* (ie, a combination of the *health nonprofit* and *government* sources in Alagha and Helbing [29]). Although the authors observed Apple Siri and Google Assistant responding and providing expert information more frequently than Amazon Alexa (in the *voice-and-display* modality only), our study showed an advantage of Google Assistant, followed by Amazon Alexa. The higher advantage of Amazon Alexa observed in our study may be explained by the time of data collection. More specifically, Alagha and Helbing [29] tested the VAs in 2018, which was before Amazon would instantiate a partnership with the NHS in 2019 to provide reliable health information [40] (see also the Principal Results section). Thus, Amazon Alexa’s ability to respond to health-related questions and the reliability of its sources may have increased since then.

#### Response Reliability Versus Response Appropriateness

Considering our results and the work of Boyd and Wilson [27] and of Alagha and Helbing [29], Google Assistant seems to be the best solution for health-related information lookup and thus for best supporting patients with NCDs. This conclusion may, however, be challenged by studies by Kocaballi et al [28] and Yang et al [31].

Kocaballi et al [28] assessed how frequently Amazon Alexa, Apple Siri, and Google Assistant would provide *appropriate* responses to safety-critical and non-safety-critical questions. Appropriateness was defined as the VA *recommending to get help from a health professional or service and to provide specific contact information* if the question was safety-critical and as *including relevant information to solve the problem* raised in the question if the question was non-safety-critical [28]. The author observed that although Google Assistant often provided a web source with its response, Apple Siri was the one providing the highest number of appropriate responses. Similarly, Yang et al [31], who assessed the *clinical appropriateness* of VAs’ responses, observed Google Assistant to provide an *appropriate* response only 21% of the time, while Amazon Alexa performed slightly better, with 29% of *appropriate* responses. *Appropriateness* was defined by 2 physicians comparing the response provided by the VA to the answer present in the American College of Obstetricians and Gynecologists patient-focused FAQs. Although *appropriateness* evaluation is more meticulous because of analyzing the content, it is more subjective. We approached the responses from a *reliability* perspective and assessed them solely on an objective level, that is, by assessing the use of recognized health web sources. Given that, as mentioned above, source type may not be sufficient to evaluate information quality, and future research should combine source evaluation with professional content evaluation to obtain a more complete representation of the VAs’ ability to provide patients with reliable information.

#### Leveraging Speech Interaction

In general, most of the related work presented above [27-29] evaluated VAs’ responses by considering both display and voice responses. That is, if the VA was not to vocally synthesize a direct response to the question but to say *Here’s what I found* and visually showed a list of results, the first of that list was still considered for evaluation. In our study, we considered only voice responses. The rationale behind this decision is that the use of VAs for (health-related) information lookup is truly advantageous and accessible if it can breach the barriers of lack of literacy [18]; visual, motor, or cognitive inabilities [19-24]; or manual unavailability [13-17]. Only Yang et al [31] reported whether the VAs provided a voice response. Although the differences between VAs were not statistically significant, the authors showed Apple Siri to be the least reliable and Amazon Alexa to be the most reliable. Our results replicate the low voice response rate of Apple Siri but show Google Assistant to respond vocally more frequently than Alexa. Given the small sample of questions and low statistical power in Yang et al [31], future research should evaluate a larger sample and contrast the results to the findings of Yang et al [31] and this study.

#### FAQ Relevance

Although the questions used in the studies discussed above were based on health-related web pages [27,29,31], none of them verified the questions’ relevance for the patients themselves in the real-world practice. In particular, gathering questions from health-related websites ensured including questions that are of most interest to the affected population, that is, not only patients but potentially also their caregivers and close social network. However, we aimed to target questions specifically relevant to the patients, as they are the protagonists of self-management. Thus, we submitted the selected questions to the respective practicing medical specialists and explicitly asked them to rate their frequency, considering the questions coming from the patients. The fact that not all questions were relevant is also supported by the fact that specialists did not select all questions as being “Often” to “Always” asked by patients (see also Multimedia Appendix 1).

### Implications

Complications related to NCDs, such as CVD, cancer, chronic respiratory disease, and DM, are among the main causes of mortality, accounting for 71% and 74% of all deaths worldwide in 2016 [1] and 2019 [2], respectively. Preventing those complications or their worsening is, therefore, crucial for survival. Engaging with self-management solutions is the best preventive practice [3,4]. VAs can support self-management through efficient and accessible information delivery by fostering patient’s health literacy [44,45] (ie, the ability to *obtain*, *process*, *understand*, *and communicate health-related information* to a level that favors positive health behavior [46]). For instance, an individual with a chronic respiratory disease who can ask their VA about smoking cessation strategies and put those into practice has higher chances to stop smoking and mitigate their health conditions. Our results show that Amazon Alexa and Google Assistant are capable of providing reliable health information through pure speech-based interaction, although such ability differs across NCDs.

### Conclusion

This study provides evidence of the ability of Vas, such as Amazon Alexa, Apple Siri, and Google Assistant, to provide reliable voice responses to questions related to NCD management compared to the standard consumer-accessible method of information lookup (ie, web search). We validated NCD-related questions with practicing medical professionals, submitted a set of 60 questions to each VA, and assessed the response rate and source type. We answered the first research question (ie, *Is the response rate dependent on developers and modality?*), observing that Google Assistant responded to most of the answers and Apple Siri responded to the fewest. Modality played a minimal role, whereas Google Assistant and Apple Siri responded slightly more often in the *voice-only* modality and Amazon Alexa in the *voice-and-display* modality. Moreover, we answered our second research question (ie, *Is the source type dependent on developers and modality?*), finding that Google Assistant based most of its responses on *expert* sources of information, even outperforming the web search snippets. Furthermore, Amazon Alexa was less reliable but provided *expert* sources more than 50% of the times. Finally, Apple Siri was the least reliable, providing a considerable percentage of *crowdsourced* sources or often not providing a source at all. Across modalities, Amazon Alexa and Apple Siri similarly provided *expert* sources, while Google Assistant did so more frequently in the *voice-only* modality. Thus, the variation seemed to be more influenced by *developer* than *modality*. Finally, answering our third and fourth research questions (ie, *Is the response rate dependent on developers and disease?* and *Is the source type dependent on developers and disease?*), we observed that although there is a slight variation across the diseases, Google Assistant showed a general clear advantage. Nevertheless, although Google Assistant seems to be a good option to ask NCD-related questions, a large number of *commercial* sources was used, in particular for COPD. Providing patients with unverified or non-evidence-based information may be counterproductive if not dangerous. Therefore, we call out health organizations to collaborate with technology companies, such as Google, Amazon, and Apple, to ensure patients with NCDs are provided with openly reliable (ie, expert) information about the management of their condition. Finally, as the algorithms behind the VAs continuously change, future research should establish the temporal consistency of these results.

To conclude, our contributions lie in the following aspects. First, to the best of our knowledge, no previous research assessed the reliability of the 3 most prevalent VAs in responding to NCD-related questions. Second, we tested the selected VAs by submitting questions that we validated with practicing medical specialists in terms of the frequency of occurrence in their medical consultations. Third, we systematically controlled for the effect of visual display by testing both smart speakers and display devices for each VA. Finally, as previous research remains rather preliminary and lacks transparent method reporting [47], we aimed to report our methods as precisely as possible in the hope of stimulating informed future research on the ability of VAs to retrieve reliable information about health-related topics.

## Appendix

Multimedia Appendix 1 Complete list of selected questions.

Multimedia Appendix 2 Complete list of the voice assistant's responses and sources.

## Abbreviations

AD: Alzheimer’s disease
CKD: chronic kidney disease
COPD: chronic obstructive pulmonary disease
CVA: cerebrovascular accident
CVD: cardiovascular disease
DM: diabetes mellitus
LCA: lung cancer
NCD: noncommunicable disease
NHS: National Health Service
VA: voice assistant
