# Supplementary material for: Reliability of Commercial Voice Assistants’ Responses to Health-Related Questions in Noncommunicable Disease Management: Factorial Experiment Assessing Response Rate and Source of Information
Source: J Med Internet Res. 2021 Dec 20;23(12):e32161. doi: 10.2196/32161 (PMC8726026; doi:10.2196/32161)
Supplement: Multimedia Appendix 1 [file jmir_v23i12e32161_app1.pdf]

Multimedia Appendix 1: Complete list of selected questions

This is a Multimedia Appendix to a full manuscript published in the J Med Internet Res. For full copyright and citation information see http://dx.doi.org/10.2196/32161

Disease	Question	Source	Rating
Cardiovascular disease	How can i reduce my cholesterol?	healthline.com	4.5
Cardiovascular disease	How long do i need to take medicine for my heart problems?	Medical specialist	5
Cardiovascular disease	How often should i take medicine for my heart problems?	Medical specialist	5
Cardiovascular disease	What are the main causes of coronary heart disease?	Replicated by authors based on [What causes type one diabetes?]	5
Cardiovascular disease	What are the side effects of my medicine for my heart problems?	Replicated by authors based on [What are the side effects of chemotherapy?]	5
Cardiovascular disease	What can i eat if my cholesterol level is high?	betterhealth.vic.gov.au	4.5
Cardiovascular disease	What causes heart problems?	Replicated by authors based on [What causes type one diabetes?]	5
Cardiovascular disease	What is a normal cholesterol level?	Adapted from aafp.org	4.5
Cardiovascular disease	What kind of exercises can i do if i have heart problems?	Medical specialist	5
Cardiovascular disease	What is a stent?	Medical specialist	4.5
Cerebrovascular disease	How can i reduce the risk of developing stroke?	hopkinsmedicine.org	5
Cerebrovascular disease	What causes a stroke?	everydayhealth.com	5
Cerebrovascular disease	How do you identify a stroke?	saebo.com	4.5
Cerebrovascular disease	What are the treatment options for stroke?	hopkinsmedicine.org	5
Cerebrovascular disease	What type of rehabilitation will i need if i had a stroke?	everydayhealth.com	4.5
Cerebrovascular disease	How long will i be in rehab if i had a stroke?	everydayhealth.com	4.5
Cerebrovascular disease	How long does it take to fully recover from a stroke?	everydayhealth.com	4.5
Cerebrovascular disease	What are my chances of having another stroke if i already had one?	everydayhealth.com	4.5
Cerebrovascular disease	How does having a stroke influence the quality of my life?	Replicated by authors based on [How does lung cancer influence the quality of my life?]	4.5
Cerebrovascular disease	What can i do to stay healthier after having a stroke?	everydayhealth.com	4.5
Chronic kidney disease	What is dialysis?	crh.org	4.5
Chronic kidney disease	Are both kidneys affected by chronic kidney disease?	webmd.com	5
Chronic kidney disease	Do i feel tired because of chronic kidney disease?	Medical specialist	5
Chronic kidney disease	Can i do a ct with contrast media if i have chronic kidney disease?	Medial specialist	5
Chronic kidney disease	What percentage of my kidney will function if i have chronic kidney disease?	Medical specialist	5
Chronic kidney disease	Is it possible to live with one functioning kidney?	healthline.com	5
Chronic kidney disease	Does chronic kidney disease make me a corona-risk patient?	Medical specialist	5
Chronic kidney disease	How much should i drink if i have chronic kidney disease?	Medical specialist	5
Chronic kidney disease	What can i eat if i have chronic kidney disease?	healthline.com	4.5
Chronic kidney disease	Can i use protein supplements if i have chronic kidney disease?	Medical specialist	5
COPD	What will happen when my COPD gets worse?	Medical specialist	5
COPD	Can i travel by plane with COPD?	Medical specialist	4.5
COPD	Do all smokers develop COPD?	Medical specialist	4.5
COPD	Does COPD lead to death?	Adapted from Medical specialist [Will I die because of not being able to breathe anymore if I have COPD?]	4.5
COPD	How can i treat COPD?	COPDfoundation.org	4.5
COPD	What are the signs that my COPD is getting worse?	Medical specialist	4.5
COPD	What causes COPD?	nhs.uk	4.5
COPD	What should i do if i have COPD and cough quite often?	Medical specialist	4.5
COPD	What is the life expectancy for a COPD patient?	templehealth.org	4
COPD	Why do i have difficulties breathing with COPD?	Medical specialist	4.5
Diabetes	What should my normal blood glucose be?	mauryregional.com	5
Diabetes	How long do i need to take my diabetes medicine?	Replicated by authors based on [How long do I need to take medicine for my heart problems?]	5
Diabetes	What are the side effects of my diabetes medicine?	Replicated by authors based on [What are the side effects of chemotherapy?]	5
Diabetes	What times of the day should i inject insulin?	Adapted from aafp.org	5
Diabetes	How do i define the amount of insulin i need?	Adapted from diabeticretinopathy.org.uk	5
Diabetes	What are the risks for my unborn child if my gestational diabetes is not well controlled?	marchofdimes.org	5
Diabetes	What are the risks of type one diabetes?	Replicated by authors based on [What are the risks of chemotherapy?]	5
Diabetes	What are possible complications of type one diabetes	Medical specialist	5
Diabetes	What can i eat if i have type one diabetes?	mauryregional.com	5
Diabetes	What can i eat if i have type two diabetes?	mauryregional.com	5
Lung cancer	How can lung cancer be treated?	webmd.com	5
Lung cancer	How does cancer spread?	Medical specialist	5
Lung cancer	How does radiation therapy work?	texasoncology.com	5
Lung cancer	How is chemotherapy administered?	texasoncology.com	5
Lung cancer	What are the side effects of chemotherapy?	cancercenter.com	5
Lung cancer	What are the side effects of radiation therapy?	cancercenter.com	5
Lung cancer	What are the stages of cancer?	foxchase.org	5
Lung cancer	What do the different stages of cancer mean?	foxchase.org	5
Lung cancer	What is radiation therapy?	cancercenter.com	5
Lung cancer	What is the goal of radiation therapy?	cancercenter.com	5
