# Supplementary material for: Reliability of Commercial Voice Assistants’ Responses to Health-Related Questions in Noncommunicable Disease Management: Factorial Experiment Assessing Response Rate and Source of Information
Source: J Med Internet Res. 2021 Dec 20;23(12):e32161. doi: 10.2196/32161 (PMC8726026; doi:10.2196/32161)
Supplement: Multimedia Appendix 2 [file jmir_v23i12e32161_app2.pdf]

Multimedia Appendix 2: Complete list of voice assistant's responses and sources

This is a Multimedia Appendix to a full manuscript published in the J Med Internet Res. For full copyright and citation information see http://dx.doi.org/10.2196/32161

		Amazon voice and display			Amazon voice only			Apple voice and display			Apple voice only			Google display only			Google voice and display			Google voice only		
Disease	Question	Response	Source	Source type	Response	Source	Source type	Response	Source	Source type	Response	Source	Source type	Response	Source	Source type	Response	Source	Source type	Response	Source	Source type
Cardiovascular disease	How can i reduce my cholesterol?	Here's something i found on the web. According to medpagetoday.com: a focus on a plant-based diet reducing saturated fat and increasing fiber with vegetables and whole grains has been shown to reduce cholesterol levels with or without the use of statins.	Medpagetoday.com	Commercial	Here's something i found on the web. According to medpagetoday.com: a focus on a plant-based diet reducing saturated fat and increasing fiber with vegetables and whole grains has been shown to reduce cholesterol levels with or without the use of statins.	Medpagetoday.com	Commercial	NA	NA	NA	NA	NA	NA	A few changes in your diet can reduce cholesterol and improve your heart health: reduce saturated fats. Saturated fats found primarily in red meat and full-fat dairy products raise your total cholesterol. ... Eliminate trans fats. ... Eat foods rich in omega-3 fatty acids. ... Increase soluble fiber. ... Add whey protein.	Mayoclinic.org	Expert	Here's a summary from mayo clinic: a few changes in your diet can reduce cholesterol and improve your heart health: 1. Reduce saturated fats. Saturated fats found primarily in red meat and full-fat dairy products raise your total cholesterol. 2. Eliminate trans fats. 3. Eat foods rich in omega-3 fatty acids. 4. Increase soluble fiber. and 5. Add whey protein.	Mayoclinic.org	Expert	Here's a summary from the website mayoclinic.org: a few changes in your diet can reduce cholesterol and improve your heart health: 1. Reduce saturated fats. Saturated fats found primarily in red meat and full-fat dairy products raise your total cholesterol. 2. Eliminate trans fats. 3. Eat foods rich in omega-3 fatty acids. 4. Increase soluble fiber. and 5. Add whey protein.	Mayoclinic.org	Expert
Cardiovascular disease	How long do i need to take medicine for my heart problems?	NA	NA	NA	NA	NA	NA	NA	NA	NA	NA	NA	NA	NA	NA	NA	NA	NA	NA	Sorry i don't have any information about that. But i found something related. Do you want to know how long does it take for heart medication to work?[Follow-up:]on the website harvard.edu they say: giving it time. Some medicines can take longer to start working. For example it might be two to four weeks before a cholesterol-lowering statin drug like atorvastatin takes effect.	Health.harvard.edu	Expert
Cardiovascular disease	How often should i take medicine for my heart problems?	NA	NA	NA	NA	NA	NA	NA	NA	NA	NA	NA	NA	NA	NA	NA	NA	NA	NA	Sorry i don't have any information about that. But i found something related. Do you want to know can you stop heart medication?[Follow-up:]on the website rochester.edu they say: never stop taking a medicine without your healthcare provider's ok. If price is an issue ask your healthcare provider if there's a lower-cost alternative. If you suddenly stop taking beta-blockers your heart rate and blood pressure can go up in the short term from a withdrawal effect.	Urmc.rochester.edu	Expert
Cardiovascular disease	What are the main causes of coronary heart disease?	According to the uk standard of care as per the nhs: coronary heart disease is the term that describes what happens when your heart's blood supply is blocked or interrupted by a build-up of fatty substances in the coronary arteries. This is caused by lifestyle factors and other conditions such as smoking high cholesterol high blood pressure and diabetes.	Nhs.uk	Expert	According to the uk standard of care as per the nhs: coronary heart disease is the term that describes what happens when your heart's blood supply is blocked or interrupted by a build-up of fatty substances in the coronary arteries. This is caused by lifestyle factors and other conditions such as smoking high cholesterol high blood pressure and diabetes.	Nhs.uk	Expert	The answer i found is arteriosclerosis.	Wikipedia.org	Crowdsourced	The answer i found is arteriosclerosis.	Not stated	Not stated	Risk factors for coronary artery disease include: age. Getting older increases your risk of damaged and narrowed arteries. Sex. Men are generally at greater risk of coronary artery disease. ... Family history. ... Smoking. ... High blood pressure. ... High blood cholesterol levels. ... Diabetes. ... Overweight or obesity.	Mayoclinic.org	Expert	According to cdc: coronary artery disease is caused by plaque buildup in the wall of the arteries that supply blood to the heart. Plaque is made up of cholesterol deposits. Plaque buildup causes the inside of the arteries to narrow over time. This process is called atherosclerosis.	Cdc.gov	Expert	On the website cdc.gov they say: coronary artery disease is caused by plaque buildup in the wall of the arteries that supply blood to the heart. Plaque is made up of cholesterol deposits. Plaque buildup causes the inside of the arteries to narrow over time. This process is called atherosclerosis.	Cdc.gov	Expert
Cardiovascular disease	What are the side effects of my medicine for my heart problems?	This might answer your question: i know about a number of medications including sertraline whose side effect is some but not all possible side effects of sertraline include nausea diarrhea drowsiness or insomnia. Some serious side effects of this drug include easy bruising or bleeding black stools abnormal heartbeat eye pain or vision changes. This drug may rarely cause worsening depression other mental or mood changes suicidal thoughts or a painful or prolonged erection. Severe allergic reactions include rash itching severe dizziness swelling or trouble breathing. For medical advice consult your healthcare professional. methen-hyosc-m.blue-sal-naphos and fat emulsion.	Not stated	Not stated	This might answer your question: i know about a number of medications including sertraline whose side effect is some but not all possible side effects of sertraline include nausea diarrhea drowsiness or insomnia. Some serious side effects of this drug include easy bruising or bleeding black stools abnormal heartbeat eye pain or vision changes. This drug may rarely cause worsening depression other mental or mood changes suicidal thoughts or a painful or prolonged erection. Severe allergic reactions include rash itching severe dizziness swelling or trouble breathing. For medical advice consult your healthcare professional. methen-hyosc-m.blue-sal-naphos and fat emulsion.	Not stated	Not stated	NA	NA	NA	NA	NA	NA	NA	NA	NA	According to rxlist: what are common side effects of coronary heart disease medications? Each type of coronary heart disease medication has different side effects. Antiplatelet drugs can cause diarrhea rash or itching abdominal pain headache chest pain muscle aches and dizziness.	Rxlist.com	Commercial	On the website rxlist.com they say: what are common side effects of coronary heart disease medications? Each type of coronary heart disease medication has different side effects. Antiplatelet drugs can cause diarrhea rash or itching abdominal pain headache chest pain muscle aches and dizziness.	Rxlist.com	Commercial
Cardiovascular disease	What can i eat if my cholesterol level is high?	Here's something i found on the web. According to nih.gov: evidence-based information on the safety and effectiveness of soy flaxseed garlic red yeast rice and other dietary supplements for high cholesterol.	Nih.gov	Expert	Here's something i found on the web. According to nih.gov: evidence-based information on the safety and effectiveness of soy flaxseed garlic red yeast rice and other dietary supplements for high cholesterol.	Nih.gov	Expert	NA	NA	NA	NA	NA	NA	Add these foods to lower ldl cholesterol: oats. ... Barley and other whole grains. ... Beans. ... Eggplant and okra. ... Nuts. ... Vegetable oils. ... Apples grapes strawberries citrus fruits. ... Foods fortified with sterols and stanols.	Health.harvard.edu	Expert	Here's a summary from harvard health - harvard university: foods that make up a low cholesterol diet can help reduce high levels. Oats. Barley and other whole grains. Beans. Eggplant and okra. Nuts. Vegetable oils. Apples grapes strawberries citrus fruits. and more.	Health.harvard.edu	Expert	I found 6 on the website www.nhs.uk. Wholemeal bread bran and wholegrain cereals. fruit and vegetables. potatoes with their skins on. oats and barley. pulses such as beans peas and lentils. and finally nuts and seeds.	Nhs.uk	Expert
Cardiovascular disease	What causes heart problems?	Here's something i found on the web. According to nih.gov: the most common cause of heart disease is narrowing or blockage of the blood vessels that supply blood to the heart.	Nih.gov	Expert	Here's something i found on the web. According to nih.gov: the most common cause of heart disease is narrowing or blockage of the blood vessels that supply blood to the heart.	Nih.gov	Expert	NA	NA	NA	NA	NA	NA	A buildup of fatty plaques in your arteries (atherosclerosis) is the most common cause of coronary artery disease. Unhealthy lifestyle habits such as a poor diet lack of exercise being overweight and smoking can lead to atherosclerosis.	Mayoclinic.org	Expert	According to mayo clinic: a buildup of fatty plaques in your arteries is the most common cause of coronary artery disease.	Mayoclinic.org	Expert	On the website mayoclinic.org they say: a buildup of fatty plaques in your arteries —atherosclerosis— is the most common cause of coronary artery disease.	Mayoclinic.org	Expert
Cardiovascular disease	What is normal cholesterol level?	According to the nih a healthy cholesterol level is under 200 mg/dl while a healthy ldl level is under 100 mg/dl and a healthy hdl level 60 mg/dl and higher.	Nih.gov	Expert	According to the nih a healthy cholesterol level is under 200 mg/dl while a healthy ldl level is under 100 mg/dl and a healthy hdl level 60 mg/dl and higher.	Nih.gov	Expert	NA	NA	NA	NA	NA	NA	[Table with good borderline high and low levels of total and ldl cholesterol]	Healthline.com	Commercial	According to medical news today: total cholesterol levels less than 200 milligrams per deciliter are considered desirable for adults. A reading between 200 and 239 mg/dl is considered borderline high and a reading of 240 mg/dl and above is considered high.	Medicalnewstoday.com	Commercial	On the website medicalnewstoday.com they say: \ldl cholesterol levels should be less than 100 mg/dl.\ Do you want a little more context?[Follow-up:]total cholesterol levels less than 200 milligrams per deciliter are considered desirable for adults. A reading between 200 and 239 mg/dl is considered borderline high and a reading of 240 mg/dl and above is considered high. Ldl cholesterol levels should be less than 100 mg/dl.	Medicalnewstoday.com	Commercial
Cardiovascular disease	What kind of exercises can i do if i have heart problems?	Here's something i found on the web. According to medscape.com: i routinely recommend that patients have a light weightlifting regimen doing such things as biceps curls bent-over rowing bent-knee sit-up and push-up exercises.	Medscape.com	Commercial	Here's something i found on the web. According to nih.gov: aerobic (or endurance) exercise uses your large muscle groups (chest legs and back) to increase your heart rate and breathing.	Nih.gov	Expert	NA	NA	NA	NA	NA	NA	Examples: brisk walking running swimming cycling playing tennis and jumping rope. Heart-pumping aerobic exercise is the kind that doctors have in mind when they recommend at least 150 minutes per week of moderate activity.	Hopkinsmedicine.org	Expert	According to johns hopkins medicine: examples: brisk walking running swimming cycling playing tennis and jumping rope. Heart-pumping aerobic exercise is the kind that doctors have in mind when they recommend at least 150 minutes per week of moderate activity.	Hopkinsmedicine.org	Expert	On the website hopkinsmedicine.org they say: examples: brisk walking running swimming cycling playing tennis and jumping rope. Heart-pumping aerobic exercise is the kind that doctors have in mind when they recommend at least 150 minutes per week of moderate activity.	Hopkinsmedicine.org	Expert
Cardiovascular disease	What should i avoid doing if i have heart problems?	NA	NA	NA	NA	NA	NA	NA	NA	NA	NA	NA	NA	Don't smoke or use tobacco. One of the best things you can do for your heart is to stop smoking or using smokeless tobacco. ... Get moving: aim for at least 30 to 60 minutes of activity daily. ... Eat a heart-healthy diet. ... Maintain a healthy weight. ... Get good quality sleep. ... Manage stress. ... Get regular health screenings.	Mayoclinic.org	Expert	Here's a summary from american heart association: 8 things you can do to prevent heart disease and stroke. Know your risk. If you're between 40 and 75 years old and have never had a heart attack or stroke use our check. Eat a healthy diet. Be physically active. Watch your weight. and more.	Heart.org	Expert	I found a list on the website heart.org. 8 things you can do to prevent heart disease and stroke. Here are the first 5. Know your risk. Eat a healthy diet. Be physically active. Watch your weight. and live tobacco-free. Want to hear more?[Follow-up:]alright. Here are the last 3: manage conditions. Take your medicine. and be a team player.	Heart.org	Expert
Cerebrovascular accident	How can i reduce the risk of developing stroke?	Here's something i found on the web. According to verywellhealth.com: lifestyle changes such as starting moderate exercise reducing stress and most importantly quitting smoking can dramatically reduce your risk of stroke.	Verywellhealth.com	Expert	Here's something i found on the web. According to verywellhealth.com: lifestyle changes such as starting moderate exercise reducing stress and most importantly quitting smoking can dramatically reduce your risk of stroke.	Verywellhealth.com	Expert	NA	NA	NA	NA	NA	NA	These are the most important steps you can take to lower your risk of stroke: keep your blood pressure in the normal range. If you smoke quit. Keep your blood sugar (glucose) in the normal range. If you have heart disease treat it. Keep your cholesterol levels in the normal range. Stay at a healthy weight. Get active.	Health.gov	Expert	Here is information from office of disease prevention and health promotion.	Health.gov	Expert	Here is some information from the web that might possibly help. On the website nhs.uk they say: the best way to help prevent a stroke is to eat a healthy diet exercise regularly and avoid smoking and drinking too much alcohol. These lifestyle changes can reduce your risk of problems like: arteries becoming clogged with fatty substances. High blood pressure.	Nhs.uk	Expert
Cerebrovascular accident	What causes a stroke?	According to mayo clinic: a stroke is caused either by a blocked artery affecting blood flow to the brain or by a blood vessel in your brain leaking or bursting. Blocked or narrowed arteries are caused by fatty deposits that build up in arteries or blood clots or other debris that go through your bloodstream and lodge in narrower brain arteries. Several things can cause leaky or ruptured blood vessels in the brain including uncontrolled high blood pressure overuse of blood thinners and weak spots in your blood vessel walls.	Mayoclinic.org	Expert	According to mayo clinic: a stroke is caused either by a blocked artery affecting blood flow to the brain or by a blood vessel in your brain leaking or bursting. Blocked or narrowed arteries are caused by fatty deposits that build up in arteries or blood clots or other debris that go through your bloodstream and lodge in narrower brain arteries. Several things can cause leaky or ruptured blood vessels in the brain including uncontrolled high blood pressure overuse of blood thinners and weak spots in your blood vessel walls.	Mayoclinic.org	Expert	Here is what i found from mayoclinic.org. There are two main causes of stroke: a blocked artery (ischemic stroke) or leaking or bursting of a blood vessel (hemorrhagic stroke).	Mayoclinic.org	Expert	Here is what i found from mayoclinic.org. There are two main causes of stroke: a blocked artery (ischemic stroke) or leaking or bursting of a blood vessel (hemorrhagic stroke). For any medical advice consult your doctor! For more say open this on iphone!	Mayoclinic.org	Expert	There are two main causes of stroke: a blocked artery (ischemic stroke) or leaking or bursting of a blood vessel (hemorrhagic stroke). Some people may have only a temporary disruption of blood flow to the brain known as a transient ischemic attack (tia) that doesn't cause lasting symptoms.	Mayoclinic.org	Expert	According to mayo clinic: there are two main causes of stroke: a blocked artery —ischemic stroke— or leaking or bursting of a blood vessel.	Mayoclinic.org	Expert	On the website mayoclinic.org they say: there are two main causes of stroke: a blocked artery —ischemic stroke— or leaking or bursting of a blood vessel.	Mayoclinic.org	Expert

Cerebrovascular accident	What are the treatment options for stroke?	According to mayo clinic: emergency treatment for stroke depends on the type of stroke. The most common kind an ischemic stroke blocks an artery. To treat it doctors must quickly restore blood flow to the brain with drugs or emergency procedures performed directly inside the blocked blood vessel. A hemorrhagic stroke involves bleeding into the brain. Emergency treatment for this type of stroke focuses on controlling the bleeding and reducing pressure in the brain. Surgery or other procedures may be needed to help reduce future risk. After emergency treatment stroke care focuses on helping you recover as much function as possible and return to independent living.	Mayoclinic.org	Expert	According to mayo clinic: emergency treatment for stroke depends on the type of stroke. The most common kind an ischemic stroke blocks an artery. To treat it doctors must quickly restore blood flow to the brain with drugs or emergency procedures performed directly inside the blocked blood vessel. A hemorrhagic stroke involves bleeding into the brain. Emergency treatment for this type of stroke focuses on controlling the bleeding and reducing pressure in the brain. Surgery or other procedures may be needed to help reduce future risk. After emergency treatment stroke care focuses on helping you recover as much function as possible and return to independent living.	Mayoclinic.org	Expert	The answer i found is pentoxifylline.	Wikipedia.org	Crowdsourced	According to wikipedia it's pentoxifylline.	Wikipedia.org	Crowdsourced	How is a stroke treated? You may get a clot-dissolving medicine called tissue plasminogen activator (tpa). ... You may also get aspirin or another antiplatelet medicine. In some cases a procedure may be done to restore blood flow.	Stanfordhealthcare.org	Expert	Here's a summary from nhs: treating ischaemic strokes. Thrombolysis – \clot buster\ medicine. Thrombectomy. Aspirin and other antiplatelets. Anticoagulants. Blood pressure medicines. Statins. and carotid endarterectomy.	Nhs.uk	Expert	I found 7 on the website www.nhs.uk. Treating ischaemic strokes. Thrombolysis – \clot buster\ medicine. Thrombectomy. Aspirin and other antiplatelets. Anticoagulants. Blood pressure medicines. Statins. and finally carotid endarterectomy.	Nhs.uk	Expert
Cerebrovascular accident	How do you identify a stroke?	Here's something i found on wikihow: step 1: understand the difference between a stroke and a mini-stroke. Step 2: look for two or more symptoms of a stroke. Step 3: do the f.	Wikihow.com	Crowdsourced	Here's something i found from wikihow: step 1: understand the difference between a stroke and a mini-stroke. Step 2: look for two or more symptoms of a stroke. Step 3: do the f. To learn more details about this and other how-to's from wikihow say open wikihow.	Wikihow.com	Crowdsourced	NA	NA	NA	NA	NA	NA	Signs of stroke in men and women. Sudden numbness or weakness in the face arm or leg especially on one side of the body. Sudden confusion trouble speaking or difficulty understanding speech. Sudden trouble seeing in one or both eyes. Sudden trouble walking dizziness loss of balance or lack of coordination.	Cdc.gov	Expert	NA	NA	NA	On the website webmd.com they say: warning signs. 1. Numbness or weakness in your face arm or leg especially on one side. 2. Confusion or trouble understanding other people. 3. Difficulty speaking. 4. Trouble seeing with one or both eyes. 5. Problems walking or staying balanced or coordinated. 6. Dizziness. and 7. Severe headache that comes on for no reason.	Webmd.com	Commercial
Cerebrovascular accident	What type of rehabilitation will i need if i had a stroke?	NA	NA	NA	NA	NA	NA	NA	NA	NA	NA	NA	NA	NA	NA	NA	According to mayo clinic: occupational therapy and speech therapy can help you with lost cognitive abilities such as memory processing problem-solving social skills judgment and safety awareness. Therapy for communication disorders. Speech therapy can help you regain lost abilities in speaking listening writing and comprehension.	Mayoclinic.org	Expert	I found 4 on the website mayoclinic.org. What's involved in stroke rehabilitation?. Motor-skill exercises.these exercises can help improve your muscle strength and coordination. Mobility training.you might learn to use mobility aids such as a walker canes wheelchair or ankle brace. Constraint-induced therapy. and finally range-of-motion therapy.	Mayoclinic.org	Expert
Cerebrovascular accident	How long will i be in rehab if i had a stroke?	NA	NA	NA	NA	NA	NA	NA	NA	NA	NA	NA	NA	Rehabilitation after a stroke begins in the hospital often within a day or two after the stroke. Rehab helps ease the transition from hospital to home and can help prevent another stroke. Recovery time after a stroke is different for everyone—it can take weeks months or even years. Risk factors: atrial fibrillation; hypercholesterolemia...	Cdc.gov	Expert	According to mayo clinic: the duration of your stroke rehabilitation depends on the severity of your stroke and related complications. Some stroke survivors recover quickly. But most need some form of long-term stroke rehabilitation lasting possibly months or years after their stroke.	Mayoclinic.org	Expert	On the website mayoclinic.org they say: the duration of your stroke rehabilitation depends on the severity of your stroke and related complications. Some stroke survivors recover quickly. But most need some form of long-term stroke rehabilitation lasting possibly months or years after their stroke.	Mayoclinic.org	Expert
Cerebrovascular accident	How long does it take to fully recover from a stroke?	Here's something i found on the web. According to nih.gov: with or without treatment most individuals begin to get better within 2 weeks after the initial onset of symptoms and most recover completely returning to normal function within 3 to 6 months.	Nih.gov	Expert	Here's something i found on the web. According to nih.gov: with or without treatment most individuals begin to get better within 2 weeks after the initial onset of symptoms and most recover completely returning to normal function within 3 to 6 months.	Nih.gov	Expert	NA	NA	NA	NA	NA	NA	The most rapid recovery usually occurs during the first three to four months after a stroke but some survivors continue to recover well into the first and second year after their stroke. Some signs point to physical therapy.	Stroke.org	Expert	Here's a summary from mayo clinic: the rate of recovery is generally greatest in the weeks and months after a stroke. There is evidence that performance can improve even 12 to 18 months after a stroke.	Mayoclinic.org	Expert	Here's what i understood from the website stroke.org: \the most rapid recovery usually occurs during the first three to four months after a stroke.\ Do you want a little more context?[Follow-up:]the most rapid recovery usually occurs during the first three to four months after a stroke but some survivors continue to recover well into the first and second year after their stroke.	Stroke.org	Expert
Cerebrovascular accident	What are my chances of having another stroke if i already had one?	NA	NA	NA	NA	NA	NA	NA	NA	NA	NA	NA	NA	Even after surviving a stroke you're not out of the woods since having one makes it a lot more likely that you'll have another. In fact of the 795000 americans who will have a first stroke this year 23 percent will suffer a second stroke. What can stroke patients do to avoid a recurrence?	Hopkinsmedicine.org	Expert	According to johns hopkins medicine: even after surviving a stroke you're not out of the woods since having one makes it a lot more likely that you'll have another. In fact of the 795000 americans who will have a first stroke this year 23 percent will suffer a second stroke.	Hopkinsmedicine.org	Expert	On the website hopkinsmedicine.org they say: \in fact of the 795000 americans who will have a first stroke this year 23 percent will suffer a second stroke.\ Do you want a little more context?[Follow-up:]even after surviving a stroke you're not out of the woods since having one makes it a lot more likely that you'll have another. In fact of the 795000 americans who will have a first stroke this year 23 percent will suffer a second stroke.	Hopkinsmedicine.org	Expert
Cerebrovascular accident	How does having a stroke influence the quality of my life?	Here's something i found on the web. According to medscape.com: in summary outcome after stroke is definitely influenced by the rehabilitation setting which often depends on the ability and availability of a patient's family to provide care.	Medscape.com	Commercial	Here's something i found on the web. According to medscape.com: in summary outcome after stroke is definitely influenced by the rehabilitation setting which often depends on the ability and availability of a patient's family to provide care.	Medscape.com	Commercial	NA	NA	NA	NA	NA	NA	With improvements in health care more people survive stroke but many have to cope with the physical psychological social and functional sequelae resulting in increased personal and public costs. Cerebral stroke causes a significant deterioration of the patient's functioning and worsening of her/his quality of life.	Ncbi.nlm.nih.gov	Expert	According to cleveland clinic's health essentials: while 63 percent reported greater physical challenges following a stroke compared to the general population 46 percent reported having increased difficulty with thinking.	Health.clevelandclinic.org	Expert	On the website ncbi.nlm.nih.gov they say: with improvements in health care more people survive stroke but many have to cope with the physical psychological social and functional sequelae resulting in increased personal and public costs.	Ncbi.nlm.nih.gov	Expert
Cerebrovascular accident	What can i do to stay healthier after having a stroke?	Here's something i found on the web. According to nih.gov: recurrent strokes are preventable through awareness and control of risk factors such as hypertension and through lifestyle changes such as healthier diets greater physical activity and smoking cessation.	Nih.gov	Expert	Here's something i found on the web. According to nih.gov: recurrent strokes are preventable through awareness and control of risk factors such as hypertension and through lifestyle changes such as healthier diets greater physical activity and smoking cessation.	Nih.gov	Expert	NA	NA	NA	NA	NA	NA	Here are some tips for what to eat and what to avoid to help you recover from a stroke. Eat whole mostly plant-based foods and lots of veggies. Avoid processed foods salt and sugar. Eat more legumes. Eat fish and poultry instead of red meat. Avoid saturated fat and snack on seeds and nuts.	Everydayhealth.com	Commercial	NA	NA	NA	Here is some information from the web that might possibly help. On the website strokefoundation.org.au they say: plenty of vegetables of different types and colours legumes and beans. Fruit. Grain foods mostly wholegrain and high fibre varieties such as breads cereals rice pasta noodles polenta couscous oats quinoa and barley. Lean meats and poultry fish eggs tofu nuts seeds legumes and beans.	Strokefoundation.org.au	Expert
Chronic kidney disease	Are both kidneys affected from chronic kidney disease?	NA	NA	NA	NA	NA	NA	NA	NA	NA	NA	NA	NA	NA	NA	NA	According to national kidney foundation: kidney disease usually affects both kidneys. If the kidneys' ability to filter the blood is seriously damaged by disease wastes and excess fluid may build up in the body.	Kidney.org	Expert	On the website kidney.org they say: kidney disease usually affects both kidneys. If the kidneys' ability to filter the blood is seriously damaged by disease wastes and excess fluid may build up in the body.	Kidney.org	Expert
Chronic kidney disease	Can i do a ct with contrast media if i have chronic kidney disease?	Here's something i found on the web. According to medscape.com: patients receiving intra-arterial contrast injections might be more likely to have comorbidity than patients undergoing intravenous contrast-enhanced ct such as chronic kidney disease which in itself are risk factors for ci-aki.	Medscape.com	Commercial	Here's something i found on the web. According to medscape.com: patients receiving intra-arterial contrast injections might be more likely to have comorbidity than patients undergoing intravenous contrast-enhanced ct such as chronic kidney disease which in itself are risk factors for ci-aki.	Medscape.com	Commercial	NA	NA	NA	NA	NA	NA	NA	NA	NA	According to radiology affiliates imaging: while the tests and contrast dyes used are generally safe the contrast eyes may lead to the development of kidney problems or cause problems in patients with kidney disease. During an mri or ct with contrast the radiology team injects contrast dye into an intravenous needle.	4rai.com	Commercial	Sorry i don't have any information about that. But i found something related. Do you want to know whether ct contrast is bad for kidneys? On the website kidney.org they say: in most cases contrast dyes used in tests such as ct and angiograms have no reported problems. About 2 percent of people receiving dyes can develop cin. However the risk for cin can increase for people with diabetes a history of heart and blood diseases and chronic kidney disease.	Kidney.org	Expert
Chronic kidney disease	Can i use protein supplements if i have chronic kidney disease?	Here's something i found on the web. According to nih.gov: when kidney function declines to the point where dialysis becomes necessary patients should include more protein in their diet because dialysis removes large amounts of protein from the blood.	Nih.gov	Expert	Here's something i found on the web. According to nih.gov: when kidney function declines to the point where dialysis becomes necessary patients should include more protein in their diet because dialysis removes large amounts of protein from the blood.	Nih.gov	Expert	NA	NA	NA	NA	NA	NA	NA	NA	NA	According to pubmed - nih: purpose of review: high-protein intake may lead to increased intraglomerular pressure and glomerular hyperfiltration. This can cause damage to glomerular structure leading to or aggravating chronic kidney disease.	Pubmed.ncbi.nlm.nih.gov	Expert	On the website pubmed.ncbi.nlm.nih.gov they say: purpose of review: high-protein intake may lead to increased intraglomerular pressure and glomerular hyperfiltration. This can cause damage to glomerular structure leading to or aggravating chronic kidney disease.	Pubmed.ncbi.nlm.nih.gov	Expert
Chronic kidney disease	Do i feel tired because of chronic kidney disease?	NA	NA	NA	NA	NA	NA	NA	NA	NA	NA	NA	NA	Our findings become imperative as fatigue is the most common physical symptom reported by kidney disease patients shown to be present in 74% of those with ckd-d [6]. Prior studies have explored associations of fatigue with clinical outcomes in patients with other chronic conditions.	Karger.com	Commercial	According to karger publishers: our findings become imperative as fatigue is the most common physical symptom reported by kidney disease patients shown to be present in 74% of those with ckd-d.	Karger.com	Commercial	On the website karger.com they say: fatigue is a common clinical manifestation in patients with chronic diseases including dialysis-dependent chronic kidney disease congestive heart failure rheumatologic diseases and recipients of hematopoietic stem cell transplant.	Karger.com	Commercial
Chronic kidney disease	Does chronic kidney disease make me a corona-risk patient?	NA	NA	NA	NA	NA	NA	NA	NA	NA	NA	NA	NA	NA	NA	NA	According to kidney care uk: being on dialysis having had a kidney transplant or having ckd stage 5 unfortunately does increase your risk of becoming severely unwell with covid-19 should you become infected.	Kidneycareuk.org	Expert	On the website kidneycareuk.org they say: being on dialysis having had a kidney transplant or having ckd stage 5 unfortunately does increase your risk of becoming severely unwell with covid-19 should you become infected.	Kidneycareuk.org	Expert

Chronic kidney disease	How much should i drink if i have chronic kidney disease?	Here's something i found on the web. According to nih.gov: drinking 2 or more colas per day was associated with increased risk of chronic kidney disease (adjusted odds ratio = 2.3; 95% confidence interval = 1.4-3.7).	Nih.gov	Expert	Here's something i found on the web. According to nih.gov: drinking 2 or more colas per day was associated with increased risk of chronic kidney disease (adjusted odds ratio = 2.3; 95% confidence interval = 1.4-3.7).	Nih.gov	Expert	NA	NA	NA	NA	NA	NA	The institute of medicine has estimated that men need approximately 13 cups (3 liters) of fluid daily and that women need approximately 9 cups (2.2 liters) of fluid daily. Less is more if you have kidney failure (a.k.a. end stage kidney disease).	Kidney.org	Expert	According to national kidney foundation: the institute of medicine has estimated that men need approximately 13 cups of fluid daily and that women need approximately 9 cups of fluid daily.	Kidney.org	Expert	On the website kidney.org they say: the institute of medicine has estimated that men need approximately 13 cups of fluid daily and that women need approximately 9 cups of fluid daily.	Kidney.org	Expert
Chronic kidney disease	Is it possible to live with one functioning kidney?	Here's something i found on the web. According to verywellhealth.com: most people have two kidneys but some people function well with only one.	Verywellhealth.com	Expert	Here's something i found on the web. According to verywellhealth.com: most people have two kidneys but some people function well with only one.	Verywellhealth.com	Expert	NA	NA	NA	NA	NA	NA	There may also be a chance of having high blood pressure later in life. However the loss in kidney function is usually very mild and life span is normal. Most people with one kidney live healthy normal lives with few problems. In other words one healthy kidney can work as well as two.	Kidney.org	Expert	According to national kidney foundation: most people who are born without a kidney lead normal healthy lives. A person may have had one kidney removed during an operation in order to treat an injury or a disease like cancer.	Kidney.org	Expert	On the website kidney.org they say: most people who are born without a kidney lead normal healthy lives. A person may have had one kidney removed during an operation in order to treat an injury or a disease like cancer.	Kidney.org	Expert
Chronic kidney disease	What can i eat if i have chronic kidney disease?	Here's something i found on the web. According to nih.gov: if you have advanced kidney disease you may need to limit foods such as bananas oranges potatoes and tomatoes and eat apples berries grapes and peaches instead.	Nih.gov	Expert	Here's something i found on the web. According to nih.gov: if you have advanced kidney disease you may need to limit foods such as bananas oranges potatoes and tomatoes and eat apples berries grapes and peaches instead.	Nih.gov	Expert	NA	NA	NA	NA	NA	NA	It's a diet rich in fruits veggies low-fat dairy products whole grains fish poultry beans seeds and nuts. It's low in sodium sugars and sweets fats and red meats. Talk to your doctor about it if you have ckd. They'll let you know if there are certain reasons you shouldn't try the dash diet.	Webmd.com	Commercial	According to american kidney fund: choose fresh or frozen vegetables instead of canned vegetables. If you do use canned vegetables drain and rinse them to remove extra salt before cooking or eating them. Avoid processed meats like ham bacon sausage and lunch meats. Munch on fresh fruits and vegetables rather than crackers or other salty snacks.	Kidneyfund.org	Expert	On the website kidneyfund.org they say: choose fresh or frozen vegetables instead of canned vegetables. If you do use canned vegetables drain and rinse them to remove extra salt before cooking or eating them. Avoid processed meats like ham bacon sausage and lunch meats. Munch on fresh fruits and vegetables rather than crackers or other salty snacks.	Kidneyfund.org	Expert
Chronic kidney disease	What is dialysis?	According to wikipedia: in medicine dialysis is the process of removing excess water solutes and toxins from the blood in people whose kidneys can no longer perform these functions naturally. This is referred to as renal replacement therapy. The first successful dialysis was performed in 1943.	Wikipedia.org	Crowdsourced	According to wikipedia: in medicine dialysis is the process of removing excess water solutes and toxins from the blood in people whose kidneys can no longer perform these functions naturally. This is referred to as renal replacement therapy. The first successful dialysis was performed in 1943.	Wikipedia.org	Crowdsourced	In medicine dialysis is the process of removing excess water solutes and toxins from the blood in people whose kidneys can no longer perform these functions naturally. Should i keep going? [Follow-up] this is referred as renal replacement therapy. The first successful dialysis was performed in 1943. Dialysis may need to be initiated when there is a sudden rapid loss of kidney function known as acute kidney injury or when a gradual decline in kidney function-chronic kidney diseases reaches stage 5.	Wikipedia.org	Crowdsourced	In medicine dialysis is the process of removing excess water solutes and toxins from the blood in people whose kidneys can no longer perform these functions naturally. Shall i continue? [Follow-up] this is referred as renal replacement therapy. The first successful dialysis was performed in 1943. Dialysis may need to be initiated when there is a sudden rapid loss of kidney function known as acute kidney injury or when a gradual decline in kidney function-chronic kidney diseases reaches stage 5.	Not stated	Not stated	NA	NA	NA	According to wikipedia: in medicine dialysis is the process of removing excess water solutes and toxins from the blood in people whose kidneys can no longer perform these functions naturally.	Wikipedia.org	Crowdsourced	According to wikipedia.com: in medicine dialysis is the process of removing excess water solutes and toxins from the blood in people whose kidneys can no longer perform these functions naturally. This is referred to as renal replacement therapy. The first successful dialysis was performed in 1943.	Wikipedia.org	Crowdsourced
Chronic kidney disease[†]	What percentage of my kidney will function if i have chronic kidney disease?	Here's something i found on the web. According to steadyhealth.com: doctor thinks that only 50% of my kidneys function.	Steadyhealth.com	Commercial	NA	NA	NA	NA	NA	NA	NA	NA	NA	What percentage of kidney function is considered kidney failure? If your kidney function drops below 15 percent of normal you are said to have kidney failure. You may have symptoms from the buildup of waste products and extra water in your body.	Niddk.nih.gov	Expert	According to national institute of diabetes and digestive and kidney diseases - nih: if your kidney function drops below 15 percent of normal you are said to have kidney failure. You may have symptoms from the buildup of waste products and extra water in your body.	Niddk.nih.gov	Expert	On the website niddk.nih.gov they say: if your kidney function drops below 15 percent of normal you are said to have kidney failure. You may have symptoms from the buildup of waste products and extra water in your body.	Niddk.nih.gov	Expert
Copd[†]	What will happen when my copd gets worse?	NA	NA	NA	NA	NA	NA	NA	NA	NA	NA	NA	NA	What happens when it suddenly gets worse? When copd gets worse it is called an exacerbation (ex-zass-er-bay-shun). During an exacerbation you may suddenly feel short of breath or your cough may get worse. You may also cough up phlegm and it may be thicker than normal or an unusual color.	Aafp.org	Expert	According to aafp: when copd gets worse it is called an exacerbation. During an exacerbation you may suddenly feel short of breath or your cough may get worse. You may also cough up phlegm and it may be thicker than normal or an unusual color.	Aafp.org	Expert	On the website aafp.org they say: when copd gets worse it is called an exacerbation. During an exacerbation you may suddenly feel short of breath or your cough may get worse. You may also cough up phlegm and it may be thicker than normal or an unusual color.	Aafp.org	Expert
Copd[†]	Can i travel by plane with copd?	Here's something i found on the web. According to medscape.com: there have been a limited number of studies investigating the actual response to air travel in passengers with copd.	Medscape.com	Commercial	Here's something i found on the web. According to medscape.com: there have been a limited number of studies investigating the actual response to air travel in passengers with copd.	Medscape.com	Commercial	NA	NA	NA	NA	NA	NA	For most passengers even those with respiratory disease air travel is safe and comfortable. Some patients with copd may be at risk but with screening these patients can be identified and most can travel safely with supplemental oxygen.	Thorax.bmj.com	Commercial	According to thorax: for most passengers even those with respiratory disease air travel is safe and comfortable. Some patients with copd may be at risk but with screening these patients can be identified and most can travel safely with supplemental oxygen.	Thorax.bmj.com	Commercial	On the website thorax.bmj.com they say: some patients with copd may be at risk but with screening these patients can be identified and most can travel safely with supplemental oxygen.	Thorax.bmj.com	Commercial
Copd[†]	Do all smokers develop copd?	NA	NA	NA	NA	NA	NA	NA	NA	NA	NA	NA	NA	Cigarette smoke and other irritants. In the vast majority of people with copd the lung damage that leads to copd is caused by long-term cigarette smoking. But there are likely other factors at play in the development of copd such as a genetic susceptibility to the disease because not all smokers develop copd .	Mayoclinic.org	Expert	According to tips from other journals - american family physician: about 10 to 15 percent of smokers develop copd but the optimal strategy to identify those most at risk is unknown.	Aafp.org	Expert	On the website mayoclinic.org they say: only some chronic smokers develop clinically apparent copd although many smokers with long smoking histories may develop reduced lung function. Some smokers develop less common lung conditions.	Mayoclinic.org	Expert
Copd[†]	Does copd lead to death?	Copd involves 3 million.	Not stated	Not stated	Copd involves 3 million.	Not stated	Not stated	NA	NA	NA	NA	NA	NA	Chronic obstructive pulmonary disease or copd is a category of conditions that includes emphysema and chronic bronchitis. Copd is a progressive condition that gets steadily worse. Over time the body becomes less able to take in enough oxygen. This can ultimately result in death.	Medicalnewstoday.com	Commercial	According to medical news today: copd is a progressive condition that gets steadily worse. Over time the body becomes less able to take in enough oxygen. This can ultimately result in death.	Medicalnewstoday.com	Commercial	On the website medicalnewstoday.com they say: copd is a progressive condition that gets steadily worse. Over time the body becomes less able to take in enough oxygen. This can ultimately result in death.	Medicalnewstoday.com	Commercial
Copd[†]	What causes copd?	According to mayo clinic: copd or chronic obstructive pulmonary disease is most often caused by smoking. Breathing in large amounts of secondhand smoke air pollution chemical fumes and dust can also cause lung damage that leads to copd.	Mayoclinic.org	Expert	According to mayo clinic: copd or chronic obstructive pulmonary disease is most often caused by smoking. Breathing in large amounts of secondhand smoke air pollution chemical fumes and dust can also cause lung damage that leads to copd.	Mayoclinic.org	Expert	I found. Dust and smoking.	Not stated	Not stated	I found. Dust and smoking.	Not stated	Not stated	What causes copd? Over time exposure to irritants that damage your lungs and airways can cause chronic obstructive pulmonary disease (copd) which includes chronic bronchitis and emphysema. The main cause of copd is smoking but nonsmokers can get copd too.	Lung.org	Expert	According to nhs: the main cause is smoking although the condition can sometimes affect people who have never smoked. The likelihood of developing copd increases the more you smoke and the longer you've smoked. Some cases of copd are caused by long-term exposure to harmful fumes or dust.	Nhs.uk	Expert	On the website nhs.uk they say: the main cause is smoking although the condition can sometimes affect people who have never smoked. The likelihood of developing copd increases the more you smoke and the longer you've smoked. Some cases of copd are caused by long-term exposure to harmful fumes or dust.	Nhs.uk	Expert
Copd[†]	What is the life expectancy for a copd patient?	Here's something i found on the web. According to reference.com: a healthy non-smoking 65-year old patient with stage 1 copd can live an average of 17 years after the diagnosis says a study by the national institutes of health.	Reference.com	Commercial	Here's something i found on the web. According to reference.com: a healthy non-smoking 65-year old patient with stage 1 copd can live an average of 17 years after the diagnosis says a study by the national institutes of health.	Reference.com	Commercial	NA	NA	NA	NA	NA	NA	Depending on the disease severity the five-year life expectancy for people with chronic obstructive pulmonary disease (copd) ranges from 40%-70%. That means 40-70 out of 100 people will be alive after five years of diagnosis of copd. Copd is a chronic gradually progressing lung disease that is not completely curable.	Medicinenet.com	Commercial	According to medicinenet: depending on the disease severity the five-year life expectancy for people with chronic obstructive pulmonary disease ranges from 40%-70%. That means 40-70 out of 100 people will be alive after five years of diagnosis of copd.	Medicinenet.com	Commercial	On the website medicinenet.com they say: \that means 40-70 out of 100 people will be alive after five years of diagnosis of copd.\ Do you want a little more context? [Yes] depending on the disease severity the five-year life expectancy for people with chronic obstructive pulmonary disease ranges from 40%-70%. That means 40-70 out of 100 people will be alive after five years of diagnosis of copd. Copd is a chronic gradually progressing lung disease that is not completely curable.	Medicinenet.com	Commercial
Copd[†]	How can i treat copd?	Here's something i found on the web. According to verywellhealth.com: inhalers are a mainstay of treatment for chronic obstructive pulmonary disease (copd).	Verywellhealth.com	Expert	Here's something i found on the web. According to verywellhealth.com: inhalers are a mainstay of treatment for chronic obstructive pulmonary disease (copd).	Verywellhealth.com	Expert	Fluticasone furoate. Indacaterol maleate. Indacaterol. Ambroxol. Formoterol. Would you like to hear the remaining five? [Yes] etofylline. Glycopyrrolate. Bambuterol. Erdosteine. L-carbocysteine.	Not stated	Not stated	Fluticasone furoate. Indacaterol maleate. Indacaterol. Ambroxol. Formoterol. Would you like to hear the remaining five? [Yes] etofylline. Glycopyrrolate. Bambuterol. Erdosteine. L-carbocysteine.	Not stated	Not stated	NA	NA	NA	NA	NA	NA	According to mayo clinic: rescue inhalers and inhaled or oral steroids can help control symptoms and minimise further damage.	Mayoclinic.org	Expert
Copd[†]	What are the signs that my copd is getting worse?	NA	NA	NA	NA	NA	NA	NA	NA	NA	NA	NA	NA	Changes in phlegm. Exacerbations could cause an increase in the amount of phlegm you produce and the color of the phlegm might change from clear to yellow or green says mccormack. According to the ats a change in phlegm (also called mucus or sputum) is often one of the first signs that your copd is getting worse.	Everydayhealth.com	Commercial	According to everyday health: exacerbations could cause an increase in the amount of phlegm you produce and the color of the phlegm might change from clear to yellow or green says mccormack. According to the ats a change in phlegm is often one of the first signs that your copd is getting worse.	Everydayhealth.com	Commercial	On the website everydayhealth.com they say: exacerbations could cause an increase in the amount of phlegm you produce and the color of the phlegm might change from clear to yellow or green says mccormack. According to the ats a change in phlegm is often one of the first signs that your copd is getting worse. People also sometimes ask me: \how do you know when copd is severe?\ Do you want to hear the answer? [Yes] on the website healthline.com they say: very severe copd. You are breathless all the time and it severely limits everyday activities such as dressing and undressing. At the most severe stage of copd quality of life is significantly reduced because of ongoing shortness of breath. Trouble breathing may even be life-threatening during some episodes.	Everydayhealth.com	Commercial

Copd[7]	What should i do if i have copd and cough quite often?	NA	NA	NA	NA	NA	NA	NA	NA	NA	NA	NA	NA	How to ease coughing with copd? How to ease a cough. Drink lots of water. This thins mucus and makes it easier to cough up. Avoid air pollution. Stay away from cigarette smoke and other things that can irritate your lungs. Don't take quick deep breaths through your mouth. Doing this after coughing can make it harder for mucus to leave the lungs.	Webmd.com	Commercial	Here is some information for how to ease coughing with copd? Here's a summary from webmd: how to ease a cough 1. Drink lots of water. 2. Avoid air pollution. and 3. Don't take quick deep breaths through your mouth.	Webmd.com	Commercial	Here is some information for how to ease coughing with copd? Here's a summary from the website webmd.com: how to ease a cough 1. Drink lots of water. 2. Avoid air pollution. and 3. Don't take quick deep breaths through your mouth. People also sometimes ask me: \why does someone with copd cough so much?\ Do you want to hear the answer? [Yes] on the website medicalnewstoday.com they say: people with copd often have enlarged mucous glands in their lungs that make excess mucus. The combination of this extra mucus and damaged ciliary glands that struggle to move it can result in increased coughing.	Webmd.com	Commercial
Copd[7,7]	Why do i have difficulties breathing with copd?	Here's something i found on the web. According to verywellhealth.com: in people with chronic obstructive pulmonary disease (copd) the diaphragm is weakened and doesn't work as well as it should during the breathing process.	Verywellhealth.com	Expert	Here's something i found on the web. According to verywellhealth.com: in people with chronic obstructive pulmonary disease (copd) the diaphragm is weakened and doesn't work as well as it should during the breathing process.	Verywellhealth.com	Expert	NA	NA	NA	NA	NA	NA	In people with copd the air sacs no longer bounce back to their original shape. The airways can also become swollen or thicker than normal and mucus production might increase. The floppy airways are partially blocked or obstructed making it even harder to get air out of the lungs.	Cdc.gov	Expert	According to cdc: in people with copd the air sacs no longer bounce back to their original shape. The airways can also become swollen or thicker than normal and mucus production might increase. The floppy airways are partially blocked or obstructed making it even harder to get air out of the lungs.	Cdc.gov	Expert	On the website cdc.gov they say: in people with copd the air sacs no longer bounce back to their original shape. The airways can also become swollen or thicker than normal and mucus production might increase. The floppy airways are partially blocked or obstructed making it even harder to get air out of the lungs. People also sometimes ask me: \can copd stop you from breathing?\ Do you want to hear the answer? [Yes] on the website clevelandclinic.org they say: breathing comes to us naturally and pretty easily. However if you have chronic obstructive pulmonary disease better known as copd you have trouble doing what connects us to life â€" you can't easily breathe. The disease can affect your airways air sacs or both.	Cdc.gov	Expert
Diabetes	How do i define the amount of insulin i need?	Here's something i found on the web. According to childrenwithdiabetes.com: i was once told that as a general rule children require one unit of insulin per kilogram of body weight per day.	Childrenwithdiabetes.com	Commercial	Here's something i found on the web. According to childrenwithdiabetes.com: i was once told that as a general rule children require one unit of insulin per kilogram of body weight per day.	Childrenwithdiabetes.com	Commercial	NA	NA	NA	NA	NA	NA	Add up all the carbohydrates in your meal. Divide the total carbohydrates by the insulin to carbohydrate ratio. The result is the amount of insulin units needed.	Choa.org	Expert	Here's a summary from diabetes education online - ucsf: basal/background insulin dose: 1. Basal/background insulin dose. 2. 500 ÷ total daily insulin dose. and 3. Correction factor = 1800 ÷total daily insulin dose = 1 unit of insulin will reduce the blood sugar so many mg/dl.	Dtc.ucsf.edu	Expert	On the website choa.org they say: add up all the carbohydrates in your meal. Divide the total carbohydrates by the insulin to carbohydrate ratio. The result is the amount of insulin units needed.	Choa.org	Expert
Diabetes	How long do i need to take my diabetes medicine?	Here's something i found on the web. According to nih.gov: some people with diabetes who take insulin need two to four shots a day to reach their blood glucose targets.	Nih.gov	Expert	Here's something i found on the web. According to nih.gov: some people with diabetes who take insulin need two to four shots a day to reach their blood glucose targets.	Nih.gov	Expert	NA	NA	NA	NA	NA	NA	NA	NA	NA	NA	NA	NA	Here's a summary from the website : evan sisson says that even with all of those factors to consider you can follow a general rule of thumb: expect to see 90 percent of the effects of most type 2 oral medications by the end of two weeks.	Diabetesforecast.org	Expert
Diabetes	What are possible complications of type one diabetes?	Here's something i found from sharecare: type 1 diabetes is usually diagnosed during childhood or adolescence. Because the body does not make insulin with type 1 diabetes you must take insulin daily via injections pills or other delivery system. Type 1 diabetes is managed with a healthy diet regular exercise staying at a normal weight and avoiding cigarette smoking.__Prevention is key with type 1 diabetes.one complication may include dental problems. You can prevent problems including tooth decay and gum disease by staying in touch with your dentist and being away of warning signs. Seeking treatment for tooth decay and gum disease is important to avoid invasive procedures.other complications may include heart disease kidney disease eye problems foot and skin infections and more.	Sharecare.com	Commercial	Here's something i found from sharecare: type 1 diabetes is usually diagnosed during childhood or adolescence. Because the body does not make insulin with type 1 diabetes you must take insulin daily via injections pills or other delivery system. Type 1 diabetes is managed with a healthy diet regular exercise staying at a normal weight and avoiding cigarette smoking.__Prevention is key with type 1 diabetes.one complication may include dental problems. You can prevent problems including tooth decay and gum disease by staying in touch with your dentist and being away of warning signs. Seeking treatment for tooth decay and gum disease is important to avoid invasive procedures.other complications may include heart disease kidney disease eye problems foot and skin infections and more.	Sharecare.com	Commercial	NA	NA	NA	NA	NA	NA	Eventually diabetes complications may be disabling or even life-threatening. Heart and blood vessel disease. ... Nerve damage (neuropathy). ... Kidney damage (nephropathy). ... Eye damage. ... Foot damage. ... Skin and mouth conditions. ... Pregnancy complications.	Mayoclinic.org	Expert	Here's a summary from mayo clinic: complications. Heart and blood vessel disease. Nerve damage. Kidney damage. Eye damage. Foot damage. Skin and mouth conditions. and pregnancy complications.	Mayoclinic.org	Expert	I found 7 on the website mayoclinic.org. Complications. Heart and blood vessel disease. Nerve damage. Kidney damage. Eye damage. Foot damage. Skin and mouth conditions. and finally pregnancy complications.	Mayoclinic.org	Expert
Diabetes	What are the risks for my unborn child if my gestational diabetes is not well controlled?	Here's something i found on the web. According to nih.gov: while gestational diabetes usually goes away after your baby is born you have a lifetime risk of developing type 2 diabetes.	Nih.gov	Expert	Here's something i found on the web. According to nih.gov: while gestational diabetes usually goes away after your baby is born you have a lifetime risk of developing type 2 diabetes.	Nih.gov	Expert	NA	NA	NA	NA	NA	NA	Blood sugar that is not well controlled in a woman with gestational diabetes can lead to problems for the pregnant woman and the baby: an extra large baby. ... C-section (cesarean section) ... High blood pressure (preeclampsia) ... Low blood sugar (hypoglycemia)	Cdc.gov	Expert	Here's a summary from cdc: an extra large baby. C-section. High blood pressure. and low blood sugar.	Cdc.gov	Expert	I found 4 on the website cdc.gov. An extra large baby. C-section. High blood pressure. And finally low blood sugar.	Cdc.gov	Expert
Diabetes	What are the risks of type one diabetes?	According to mayo clinic: risk factors for type 1 diabetes include having a parent or sibling with type 1 diabetes or having certain genes associated with the disease. Where you live also may play a role. Type 1 diabetes is more common in places that are farther from the equator. Although type 1 diabetes can appear at any age it tends to peak between 4 and 7 years old and between 10 and 14 years old.	Mayoclinic.org	Expert	According to mayo clinic: risk factors for type 1 diabetes include having a parent or sibling with type 1 diabetes or having certain genes associated with the disease. Where you live also may play a role. Type 1 diabetes is more common in places that are farther from the equator. Although type 1 diabetes can appear at any age it tends to peak between 4 and 7 years old and between 10 and 14 years old.	Mayoclinic.org	Expert	NA	NA	NA	Risk factors for controlled type one diabetes mellitus include family history.	Not stated	Not stated	Eventually diabetes complications may be disabling or even life-threatening. Heart and blood vessel disease. ... Nerve damage (neuropathy). ... Kidney damage (nephropathy). ... Eye damage. ... Foot damage. ... Skin and mouth conditions. ... Pregnancy complications.	Mayoclinic.org	Expert	Here's a summary from mayo clinic: eventually diabetes complications may be disabling or even life-threatening. Heart and blood vessel disease. Nerve damage. Kidney damage. Eye damage. Foot damage. Skin and mouth conditions. and pregnancy complications.	Mayoclinic.org	Expert	I found 7 on the website mayoclinic.org. Eventually diabetes complications may be disabling or even life-threatening. Heart and blood vessel disease. Nerve damage. Kidney damage. Eye damage. Foot damage. Skin and mouth conditions. and finally pregnancy complications.	Mayoclinic.org	Expert
Diabetes	What are the side effects of my diabetes medicine?	Here's something i found on reference.com: if left untreated or improperly controlled type 2 diabetes can lead to life-threatening complications such as neuropathy heart attack high blood pressure stroke or glaucoma states the mayo clinic. Nerve damage is common in patients with high blood sugar levels and can cause numbness in the limbs.	Reference.com	Commercial	Here's something i found on reference.com: if left untreated or improperly controlled type 2 diabetes can lead to life-threatening complications such as neuropathy heart attack high blood pressure stroke or glaucoma states the mayo clinic. Nerve damage is common in patients with high blood sugar levels and can cause numbness in the limbs.	Reference.com	Commercial	NA	NA	NA	NA	NA	NA	The most common side effect is low blood sugar. You may also have a wide variety of symptoms such as headaches rashes dizziness anxiety cough and dry mouth. Talk to your doctor. Some may go away as your body gets used to the medicine.	Webmd.com	Commercial	According to webmd: the most common side effect is low blood sugar. You may also have a wide variety of symptoms such as headaches rashes dizziness anxiety cough and dry mouth. Talk to your doctor. Some may go away as your body gets used to the medicine.	Webmd.com	Commercial	On the website webmd.com they say: what are the side effects? You may have nausea gas bloating diarrhea b12 deficiency and an upset stomach. These problems usually go away in a few weeks as your body gets used to the medicine.	Webmd.com	Commercial
Diabetes	What can i eat if i have type one diabetes?	Here's something i found on the web. According to nih.gov: depending on your diabetes medicines or type of insulin you may need to eat the same amount of carbohydrates at the same time each day.	Nih.gov	Expert	Here's something i found on the web. According to nih.gov: depending on your diabetes medicines or type of insulin you may need to eat the same amount of carbohydrates at the same time each day.	Nih.gov	Expert	NA	NA	NA	NA	NA	NA	Diabetes 'super foods'. Beans. Dark green leafy vegetables. Citrus fruit. Sweet potatoes. Berries. Tomatoes. Fish high in omega-3 fatty acids (like salmon) whole grains.	Webmd.com	Commercial	Here's a summary from webmd: diabetes 'super foods'. Beans. Dark green leafy vegetables. Citrus fruit. Sweet potatoes. Berries. Tomatoes. Fish high in omega-3 fatty acids. Whole grains. and more.	Webmd.com	Commercial	I found 10 on the website webmd.com. Diabetes 'super foods'. Here are the first 5. Beans. Dark green leafy vegetables. Citrus fruit. Sweet potatoes. and berries. Do you want to hear more?[Follow-up:]okay. Here's 3 more: tomatoes. Fish high in omega-3 fatty acids. and whole grains. To see the whole list follow the link i just sent to your phone.	Webmd.com	Commercial
Diabetes	What can i eat if i have type two diabetes?	Here's something i found on the web. According to nih.gov: in individuals with type 2 diabetes ingested protein can increase insulin response without increasing plasma glucose concentrations.	Nih.gov	Expert	Here's something i found on the web. According to nih.gov: in individuals with type 2 diabetes ingested protein can increase insulin response without increasing plasma glucose concentrations.	Nih.gov	Expert	NA	NA	NA	NA	NA	NA	Foods to eat for a type 2 diabetic diet meal plan include complex carbohydrates such as brown rice whole wheat quinoa oatmeal fruits vegetables beans and lentils. Foods to avoid include simple carbohydrates which are processed such as sugar pasta white bread flour and cookies pastries. Treatments: anti-diabetic medication; insulin	Medicinenet.com	Commercial	According to nhs: eat a wide range of foods – including fruit vegetables and some starchy foods like pasta. keep sugar fat and salt to a minimum. eat breakfast lunch and dinner every day – do not skip meals.	Nhs.uk	Expert	On the website nhs.uk they say: eat a wide range of foods – including fruit vegetables and some starchy foods like pasta. keep sugar fat and salt to a minimum. eat breakfast lunch and dinner every day – do not skip meals.	Nhs.uk	Expert
Diabetes	What should my normal blood glucose be?	According to the united states food and drug administration the normal blood glucose level ranges from 80 to 120 milligrams per deciliter.	Fda.gov	Expert	According to the united states food and drug administration the normal blood glucose level ranges from 80 to 120 milligrams per deciliter.	Fda.gov	Expert	NA	NA	NA	NA	NA	NA	A blood sugar level less than 140 mg/dl (7.8 mmol/l) is normal. A reading of more than 200 mg/dl (11.1 mmol/l) after two hours indicates diabetes. A reading between 140 and 199 mg/dl (7.8 mmol/l and 11.0 mmol/l) indicates prediabetes. Includes diseases: diabetes mellitus type 1treatments: exercise; insulin (medication)	Mayoclinic.org	Expert	According to mayo clinic: a blood sugar level less than 140 mg/dl is normal. A reading of more than 200 mg/dl after two hours indicates diabetes. A reading between 140 and 199 mg/dl indicates prediabetes.	Mayoclinic.org	Expert	On the website webmd.com they say: \normal blood sugar levels are less than 100 mg/dl after not eating (fasting) for at least eight hours.\ Do you want a little more context?[Follow-up:]normal blood sugar levels are less than 100 mg/dl after not eating for at least eight hours. And they're less than 140 mg/dl two hours after eating. During the day levels tend to be at their lowest just before meals.	Webmd.com	Commercial

Diabetes	What times of the day should i inject insulin?	NA	NA	NA	NA	NA	NA	NA	NA	NA	NA	NA	NA	For mealtime insulin it's best to consistently use the same part of the body for each meal. For example you can inject in your stomach prior to breakfast your thigh prior to lunch and your arm prior to dinner.	Healthline.com	Commercial	According to american diabetes association: timing. Insulin shots are most effective when you take them so that insulin goes to work when glucose from your food starts to enter your blood. For example regular insulin works best if you take it 30 minutes before you eat.	Diabetes.org	Expert	On the website diabetes.org they say: for example giving your before-breakfast insulin injection in the abdomen and your before-supper insulin injection in the leg each day give more similar blood sugar results. If you inject insulin near the same place each time hard lumps or extra fatty deposits may develop.	Diabetes.org	Expert
Lung cancer	How can lung cancer be treated?	According to mayo clinic: treatment for lung cancer may include surgery to remove the cancer along with a small amount of surrounding healthy tissue. If you have a larger lung cancer your doctor may recommend chemotherapy or radiation before surgery to shrink the cancer. Chemotherapy uses drugs to kill cancer cells. Radiation therapy uses high-powered energy beams to kill cancer cells. For advanced lung cancer immunotherapy may be an option. It uses your immune system to fight cancer. Your care team may also use medications or other treatments to help keep you comfortable if you pain or shortness of breath.	Mayoclinic.org	Expert	According to mayo clinic: treatment for lung cancer may include surgery to remove the cancer along with a small amount of surrounding healthy tissue. If you have a larger lung cancer your doctor may recommend chemotherapy or radiation before surgery to shrink the cancer. Chemotherapy uses drugs to kill cancer cells. Radiation therapy uses high-powered energy beams to kill cancer cells. For advanced lung cancer immunotherapy may be an option. It uses your immune system to fight cancer. Your care team may also use medications or other treatments to help keep you comfortable if you pain or shortness of breath.	Mayoclinic.org	Expert	Typical treatments of cancer of the trachea bronchus and lung include: surgery chemotherapy and radiation therapy.	Not stated	Not stated	Typical treatments of cancer of the trachea bronchus and lung include: surgery chemotherapy and radiation therapy.	Not stated	Not stated	People with non-small cell lung cancer can be treated with surgery chemotherapy radiation therapy targeted therapy or a combination of these treatments. People with small cell lung cancer are usually treated with radiation therapy and chemotherapy. Surgery.	Cdc.gov	Expert	According to cdc: people with non-small cell lung cancer can be treated with surgery chemotherapy radiation therapy targeted therapy or a combination of these treatments. People with small cell lung cancer are usually treated with radiation therapy and chemotherapy.	Cdc.gov	Expert	On the website cdc.gov they say: \people with small cell lung cancer are usually treated with radiation therapy and chemotherapy.\ Do you want a little more context?[Follow-up:]people with non-small cell lung cancer can be treated with surgery chemotherapy radiation therapy targeted therapy or a combination of these treatments. People with small cell lung cancer are usually treated with radiation therapy and chemotherapy. Surgery.	Cdc.gov	Expert
Lung cancer	How does cancer spread?	Here's something i found on the web. According to verywellhealth.com: cancer can spread locally via the lymphatic vessels through the bloodstream and in the case of lung cancer through the airways.	Verywellhealth.com	Expert	Here's something i found on the web. According to verywellhealth.com: cancer can spread locally via the lymphatic vessels through the bloodstream and in the case of lung cancer through the airways.	Verywellhealth.com	Expert	Here is an answer from healthline.com. Cancer cells form when dna abnormalities cause a gene to behave differently than it should. They can grow into nearby tissue spread through the bloodstream or lymph system and spread through the body.	Healthline.com	Commercial	Here is what i found from healthline.com. Cancer cells form when dna abnormalities cause a gene to behave differently than it should. They can grow into nearby tissue spread through the bloodstream or lymph system and spread through the body. For any medical advice consult your doctor!	Healthline.com	Commercial	In metastasis cancer cells break away from where they first formed (primary cancer) travel through the blood or lymph system and form new tumors (metastatic tumors) in other parts of the body. The metastatic tumor is the same type of cancer as the primary tumor.	Cancer.gov	Expert	According to national cancer institute: in metastasis cancer cells break away from where they first formed travel through the blood or lymph system and form new tumors in other parts of the body.	Cancer.gov	Expert	On the website cancer.gov they say: in metastasis cancer cells break away from where they first formed travel through the blood or lymph system and form new tumors in other parts of the body.	Cancer.gov	Expert
Lung cancer	How does radiation therapy work?	Radiation therapy or radiotherapy often abbreviated rt rtx or xrt is a therapy using ionizing radiation generally as part of cancer treatment to control or kill malignant cells and normally delivered by a linear accelerator. Radiation therapy may be curative in a number of types of cancer if they are localized to one area of the body.	Not stated	Not stated	Radiation therapy or radiotherapy often abbreviated rt rtx or xrt is a therapy using ionizing radiation generally as part of cancer treatment to control or kill malignant cells and normally delivered by a linear accelerator. Radiation therapy may be curative in a number of types of cancer if they are localized to one area of the body.	Not stated	Not stated	NA	NA	NA	NA	NA	NA	At high doses radiation therapy kills cancer cells or slows their growth by damaging their dna. Cancer cells whose dna is damaged beyond repair stop dividing or die. When the damaged cells die they are broken down and removed by the body. Radiation therapy does not kill cancer cells right away.	Cancer.gov	Expert	According to american lung association: lung cancer radiation therapy uses powerful high-energy x-rays to kill cancer cells or keep them from growing. Radiation may come from outside the body or from radioactive materials placed directly inside the lung cancer tumor.	Lung.org	Expert	On the website cancer.gov they say: \at high doses radiation therapy kills cancer cells or slows their growth by damaging their dna.\ Do you want a little more context?[Follow-up:]at high doses radiation therapy kills cancer cells or slows their growth by damaging their dna. Cancer cells whose dna is damaged beyond repair stop dividing or die. When the damaged cells die they are broken down and removed by the body. Radiation therapy does not kill cancer cells right away.	Cancer.gov	Expert
Lung cancer	How is chemotherapy administered?	According to an alexa answers contributor: chemotherapy maybe administered through pills injection and radiation; working it's best when accompanied with the supplementation of fermented wheat germ extract.	Alexaanswers.amazon.com	Crowdsourced	According to an alexa answers contributor: chemotherapy maybe administered through pills injection and radiation; working it's best when accompanied with the supplementation of fermented wheat germ extract.	Alexaanswers.amazon.com	Crowdsourced	Here is what i found from emedicinehealth.com. Chemotherapy may be given in many ways such as injection into muscles intra-arterial intraperitoneal intravenous topically or orally; some chemotherapy may be administered through specialized ports and/or pumps.	Emedicinehealth.com (owned by webmd)	Commercial	Here is what i found from emedicinehealth.com. Chemotherapy may be given in many ways such as injection into muscles intra-arterial intraperitoneal intravenous topically or orally; some chemotherapy may be administered through specialized ports and/or pumps. For any medical advice consult your doctor!	Emedicinehealth.com (owned by webmd)	Commercial	Chemotherapy is most often given as an infusion into a vein (intravenously). The drugs can be given by inserting a tube with a needle into a vein in your arm or into a device in a vein in your chest. Chemotherapy pills. Some chemotherapy drugs can be taken in pill or capsule form.	Mayoclinic.org	Expert	According to mayo clinic: chemotherapy is most often given as an infusion into a vein. The drugs can be given by inserting a tube with a needle into a vein in your arm or into a device in a vein in your chest.	Mayoclinic.org	Expert	On the website mayoclinic.org they say: chemotherapy is most often given as an infusion into a vein. The drugs can be given by inserting a tube with a needle into a vein in your arm or into a device in a vein in your chest.	Mayoclinic.org	Expert
Lung cancer	What are the side effects of chemotherapy?	According to mayo clinic: common risks of using chemotherapy drugs include nausea vomiting diarrhea hair loss fever and loss of appetite. You might feel very tired bruise easily and have mouth sores pain and constipation. Many side effects can be prevented or treated and most go away after treatment ends. Chemotherapy drugs can cause side effects that don't appear until months or years after treatment. They can include damaged lung tissue heart problems infertility kidney problems nerve damage called peripheral neuropathy and risk of a second cancer.	Mayoclinic.org	Expert	According to mayo clinic: common risks of using chemotherapy drugs include nausea vomiting diarrhea hair loss fever and loss of appetite. You might feel very tired bruise easily and have mouth sores pain and constipation. Many side effects can be prevented or treated and most go away after treatment ends. Chemotherapy drugs can cause side effects that don't appear until months or years after treatment. They can include damaged lung tissue heart problems infertility kidney problems nerve damage called peripheral neuropathy and risk of a second cancer.	Mayoclinic.org	Expert	NA	NA	NA	NA	NA	NA	Here's a list of many of the common side effects but it's unlikely you'll have all of these. Tiredness. Tiredness (fatigue) is one of the most common side effects of chemotherapy. ... Feeling and being sick. ... Hair loss. ... Infections. ... Anaemia. ... Bruising and bleeding. ... Sore mouth. ... Loss of appetite.	Nhs.uk	Expert	Here's a summary from nhs: tiredness. Feeling and being sick. Hair loss. Infections. Anaemia. Bruising and bleeding. Sore mouth. Loss of appetite. and more.	Nhs.uk	Expert	I found 15 on the website www.nhs.uk. Here are the first 5. Tiredness. Tiredness is one of the most common side effects of chemotherapy. Feeling and being sick. Hair loss. Infections. and anaemia. Do you want to hear more?[Follow-up:]sure. Here's 3 more: bruising and bleeding. Sore mouth. and loss of appetite. To see the whole list follow the link i just sent to your phone.	Nhs.uk	Expert
Lung cancer	What are the side effects of radiation therapy?	According to mayo clinic: radiation therapy side effects depend on how much radiation is used and the part of your body that's exposed. For example head and neck radiation can cause dry mouth sore throat and difficulty swallowing. Radiation to the belly can cause nausea vomiting and diarrhea. Other side effects include tiredness and hair loss and skin irritation at the treatment site. You may have no side effects or you may have several. Most can be controlled and disappear with time. Some side effects may develop later like a new cancer that's different from the first one treated with radiation.	Mayoclinic.org	Expert	According to mayo clinic: radiation therapy side effects depend on how much radiation is used and the part of your body that's exposed. For example head and neck radiation can cause dry mouth sore throat and difficulty swallowing. Radiation to the belly can cause nausea vomiting and diarrhea. Other side effects include tiredness and hair loss and skin irritation at the treatment site. You may have no side effects or you may have several. Most can be controlled and disappear with time. Some side effects may develop later like a new cancer that's different from the first one treated with radiation.	Mayoclinic.org	Expert	NA	NA	NA	NA	NA	NA	Radiation therapy aimed at a person's head or neck may cause these side effects: dry mouth. Mouth and gum sores. Difficulty swallowing. Stiffness in the jaw. Nausea. Hair loss. A type of swelling called lymphedema. Tooth decay.	Cancer.net	Expert	According to cedars-sinai: radiation-induced vasculopathy can also result in sudden bleeding in the brain known as a hemorrhagic stroke. Symptoms of a hemorrhagic stroke include: numbness weakness or inability to move of the face arm or leg especially on one side of the body.	Cedars-sinai.org	Expert	I found 6 on the website cancer.gov. Treatment areas and possible side effects. Fatigue. Hair loss. Nausea and vomiting. Skin changes. Headache. and finally blurry vision.	Cancer.gov	Expert
Lung cancer	What are the stages of cancer?	Here's what i found on reference.com: most types of cancer are classed as belonging to stages 0 i ii iii or iv. The stage assigned to the cancer refers to the extent of its spread at the time of diagnosis. According to cancer.net which stage a cancer is at determines the recommended course of treatment and the likely prognosis for the patient.	Reference.com	Commercial	Here's what i found on reference.com: most types of cancer are classed as belonging to stages 0 i ii iii or iv. The stage assigned to the cancer refers to the extent of its spread at the time of diagnosis. According to cancer.net which stage a cancer is at determines the recommended course of treatment and the likely prognosis for the patient.	Reference.com	Commercial	Cancer staging is the process of determining the extent to which a cancer has developed by growing and spreading. Shall i continue? [Follow-up] contemporary practice is to assign a number from i to iv to a cancer with i being an isolated cancer and iv being a cancer that has spread to the limit of what the assessment measures.	Wikipedia.org	Crowdsourced	Cancer staging is the process of determining the extent to which a cancer has developed by growing and spreading. That was from wikipedia. Shall i continue? [Follow-up] contemporary practice is to assign a number from i to iv to a cancer with i being an isolated cancer and iv being a cancer that has spread to the limit of what the assessment measures.	Wikipedia.org	Crowdsourced	Staging groups: stage 0 means there's no cancer only abnormal cells with the potential to become cancer. ... Stage i means the cancer is small and only in one area. ... Stage ii and iii mean the cancer is larger and has grown into nearby tissues or lymph nodes. Stage iv means the cancer has spread to other parts of your body.	Webmd.com	Commercial	According to wikipedia: cancer staging is the process of determining the extent to which a cancer has developed by growing and spreading.	Wikipedia.org	Crowdsourced	According to wikipedia.com: cancer staging is the process of determining the extent to which a cancer has developed by growing and spreading. Contemporary practice is to assign a number from i to iv to a cancer with i being an isolated cancer and iv being a cancer that has spread to the limit of what the assessment measures.	Wikipedia.org	Crowdsourced
Lung cancer	What do the different stages of cancer mean?	Here's something i found on the web. According to blurtit.com: stages are number 1 through 4 with stage one meaning that the cancer is only in the cervix and has not spread anywhere outside of the primary source through to stage 4 which means the cancer is advanced and has spread to other organs outside of the cervix.	Blurtit.com	Crowdsourced	Here's something i found on the web. According to blurtit.com: stages are number 1 through 4 with stage one meaning that the cancer is only in the cervix and has not spread anywhere outside of the primary source through to stage 4 which means the cancer is advanced and has spread to other organs outside of the cervix.	Blurtit.com	Crowdsourced	Here is an answer from wikipedia.com.org. Cancers are classified at different stages depending on how advanced tumors have progressed. Staging describes the extent or severity of an individual's cancer based on the extent of the original (primary) tumor and the extent of spread in the body.	Wikipedia.org	Crowdsourced	Here is an answer from wikipedia.org. Cancers are classified at different stages depending on how advanced tumors have progressed. Staging describes the extent or severity of an individual's cancer based on the extent of the original (primary) tumor and the extent of spread in the body. For any medical advice consult your doctor!	Wikipedia.org	Crowdsourced	Stage i means the cancer is small and only in one area. This is also called early-stage cancer. Stage ii and iii mean the cancer is larger and has grown into nearby tissues or lymph nodes. Stage iv means the cancer has spread to other parts of your body.	Webmd.com	Commercial	According to webmd: this is also called early-stage cancer. Stage ii and iii mean the cancer is larger and has grown into nearby tissues or lymph nodes. Stage iv means the cancer has spread to other parts of your body.	Webmd.com	Commercial	On the website nhs.uk they say: stage ii – the cancer has grown but hasn't spread. stage iii – the cancer is larger and may have spread to the surrounding tissues and/or the lymph nodes.	Nhs.uk	Expert
Lung cancer	What is radiation therapy?	Radiation therapy is a cancer treatment that uses intense energy beams like x-rays to kill cancer cells. Radiation therapy usually refers to treatment with high-energy beams that come from a machine outside of your body. In another type of radiation treatment brachytherapy radiation is placed inside your body. Radiation therapy damages cells by destroying the genetic material that controls how cells grow and divide. Both healthy and cancerous cells are damaged by radiation therapy but the goal is to destroy as few normal healthy cells as possible.	Not stated	Not stated	Radiation therapy is a cancer treatment that uses intense energy beams like x-rays to kill cancer cells. Radiation therapy usually refers to treatment with high-energy beams that come from a machine outside of your body. In another type of radiation treatment brachytherapy radiation is placed inside your body. Radiation therapy damages cells by destroying the genetic material that controls how cells grow and divide. Both healthy and cancerous cells are damaged by radiation therapy but the goal is to destroy as few normal healthy cells as possible.	Not stated	Not stated	Radiation therapy or radiotherapy often abbreviated rt rtx or xrt is a therapy using ionizing radiation generally as part of cancer treatment to control or kill malignant cells and normally delivered by a linear accelerator. Would you like to hear more? [Follow-up] radiation therapy may be curative in a number of types of cancer if they are localized to one area of the body. It may also be used as part of adjuvant therapy to prevent tumor recurrence after surgery to remove a primary malignant tumor.	Wikipedia.org	Crowdsourced	Radiation therapy or radiotherapy often abbreviated rt rtx or xrt is a therapy using ionizing radiation generally as part of cancer treatment to control or kill malignant cells and normally delivered by a linear accelerator. Do you want me to keep reading? [Follow-up] radiation therapy may be curative in a number of types of cancer if they are localized to one area of the body. It may also be used as part of adjuvant therapy to prevent tumor recurrence after surgery to remove a primary malignant tumor.	Not stated	Not stated	NA	NA	NA	According to wikipedia: radiation therapy or radiotherapy often abbreviated rt rtx or xrt is a therapy using ionizing radiation generally as part of cancer treatment to control or kill malignant cells and normally delivered by a linear accelerator.	Wikipedia.org	Crowdsourced	According to wikipedia.com: radiation therapy or radiotherapy often abbreviated rt rtx or xrt is a therapy using ionizing radiation generally as part of cancer treatment to control or kill malignant cells and normally delivered by a linear accelerator.	Wikipedia.org	Crowdsourced
Lung cancer	What is the goal of radiation therapy?	Here's something i found on the web. According to nih.gov: the goal of radiation therapy is to eradicate tumor burden in a manner that maintains visual function and preserves surrounding sensitive ocular tissues.	Nih.gov	Expert	Here's something i found on the web. According to nih.gov: the goal of radiation therapy is to eradicate tumor burden in a manner that maintains visual function and preserves surrounding sensitive ocular tissues.	Nih.gov	Expert	NA	NA	NA	NA	NA	NA	What are the goals of radiation therapy? Radiation oncologists use radiation therapy to destroy cancer cells and slow tumor growth while limiting the harm to nearby healthy tissue. Sometimes doctors recommend radiation therapy as the first cancer treatment.	Cancer.net	Expert	According to cancer.net: what are the goals of radiation therapy? Radiation oncologists use radiation therapy to destroy cancer cells and slow tumor growth while limiting the harm to nearby healthy tissue.	Cancer.net	Expert	On the website cancer.net they say: what are the goals of radiation therapy? Radiation oncologists use radiation therapy to destroy cancer cells and slow tumor growth while limiting the harm to nearby healthy tissue.	Cancer.net	Expert

[1] Chronic obstructive pulmonary disease
